# Design, Synthesis, and In Vitro and In Vivo Bioactivity Studies of Hydrazide–Hydrazones of 2,4-Dihydroxybenzoic Acid

**DOI:** 10.3390/ijms242417481

**Published:** 2023-12-14

**Authors:** Łukasz Popiołek, Monika Gawrońska-Grzywacz, Aleksandra Dziduch, Anna Biernasiuk, Iwona Piątkowska-Chmiel, Mariola Herbet

**Affiliations:** 1 Chair and Department of Organic Chemistry, Faculty of Pharmacy, Medical University of Lublin, 4A Chodźki Street, 20-093 Lublin, Poland; 54134@student.umlub.pl; 2 Chair and Department of Toxicology, Faculty of Pharmacy, Medical University of Lublin, 8B Jaczewskiego Street, 20-090 Lublin, Poland; monika.gawronska-grzywacz@umlub.pl (M.G.-G.); iwona.piatkowska-chmiel@umlub.pl (I.P.-C.); mariola.herbet@umlub.pl (M.H.); 3 Chair and Department of Pharmaceutical Microbiology, Faculty of Pharmacy, Medical University of Lublin, 1 Chodźki Street, 20-093 Lublin, Poland; anna.biernasiuk@umlub.pl

**Keywords:** hydrazide–hydrazone, biological activity, antibacterial activity, antifungal activity, antiproliferative activity

## Abstract

In this research, twenty-four hydrazide–hydrazones of 2,4-dihydroxybenzoic acid were designed, synthesized, and subjected to in vitro and in vivo bioactivity studies. The chemical structure of the obtained compounds was confirmed by spectral methods. Antimicrobial activity screening was performed against a panel of microorganisms for all synthesized hydrazide–hydrazones. The performed assays revealed the interesting antibacterial activity of a few substances against Gram-positive bacterial strains including MRSA—*Staphylococcus aureus* ATCC 43300 (compound **18**: 2,4-dihydroxy-*N*-[(2-hydroxy-3,5-diiodophenyl)methylidene]benzohydrazide—Minimal Inhibitory Concentration, MIC = 3.91 µg/mL). In addition, we performed the in vitro screening of antiproliferative activity and also assessed the acute toxicity of six hydrazide–hydrazones. The following human cancer cell lines were used: 769-P, HepG2, H1563, and LN-229, and the viability of the cells was assessed using the MTT method. The HEK-293 cell line was used as a reference line. The toxicity was tested in vivo on *Danio rerio* embryos using the Fish Embryo Acute Toxicity (FET) test procedure according to OECD No. 236. The inhibitory concentration values obtained in the in vitro test showed that *N*-[(4-nitrophenyl)methylidene]-2,4-dihydroxybenzhydrazide (**21**) inhibited cancer cell proliferation the most, with an extremely low IC_50_ (Inhibitory Concentration) value, estimated at 0.77 µM for LN-229. In addition, each of the compounds tested was selective against cancer cell lines. The compounds with a nitrophenyl substituent were the most promising in terms of inhibition cancer cell proliferation. The toxicity against zebrafish embryos and larvae was also very low or moderate.

## 1. Introduction

The ever-growing resistance of microorganisms to the currently used antimicrobial agents is the main reason to search for novel compounds with different chemical structures and enhanced antimicrobial properties to treat bacterial and fungal infections [1,2]. Additionally, according to the report published in 2020 by the World Health Organization (WHO), after cardiovascular disease, cancer is the second highest reason for death caused by non-communicable diseases in the world. Despite the fact that the number of cases, mortality, and types of the most frequently diagnosed cancers are not the same in all countries, which is the result of differences in the operation of healthcare systems, preventive measures, living standards, geographical location, or culture of societies, cancer is a global problem [3]. The International Agency for Research on Cancer (IARC) published statistics, according to which the most common cancers in the world in 2020 were breast, lung, and colon cancer, and the highest mortality was recorded for lung, colon, and liver cancer. In 2020, more than 19 million new cases and almost 10 million deaths due to cancer were registered. The IARC, taking into account demographic growth, gave an estimation, according to which, in 2040, these numbers will increase to 30.2 million new cases and 16.3 million deaths [4].

Lung cancer (LC) was the leading cause of cancer death in males and the second highest in females worldwide in 2020. In the case of incidence rates, LC was the most common cancer in men and third, after breast cancer and colorectum cancer, in women [4]. The available research results prove that there is an increasing trend in LC in women and, still, a large proportion of patients are diagnosed at the metastatic or disseminated stage. The prospective studies conducted every 10 years since 2000 have shown significant changes in tumor and patient characteristics. Compared with previous cohorts, in 2020, the proportion of non-smokers enhanced, and also, adenocarcinoma incidence rose [5]. In the United States of America, the 5-year relative survival rate from 2012 to 2018 for patients with lung cancer was 23% [6]. Lung adenocarcinoma is a type of non-smallcell lung cancer (NSCLC) that accounts for about 40% of all lung cancers and affects both smokers and non-smokers [7]. NSCLC is usually less sensitive to both chemotherapy and radiation therapy than small cell lung cancer is (SCLC). In the case of resectable disease, the method of choice is surgical treatment, sometimes followed by chemotherapy. However, in patients with unresectable disease, radiation therapy is used, but better results are achieved when radiation therapy is combined with chemotherapy. Patients with advanced metastatic disease can achieve better survival and symptom relief with chemotherapy and targeted drugs [7].

Although lung cancer is one of the most commonly diagnosed cancers, glioblastoma multiforme (GBM) is one of the most aggressive malignancies. Its median overall survival is estimated at approximately 15 months, remaining poor despite medical advances. GBM is a higher-grade primary brain tumor which invades the nearby brain tissue but generally does not spread to distant organs due to the existence of the blood–brain barrier. This malignant neoplasm is significantly more commonly diagnosed in men, and the median age of diagnosis is 64 years. However, the risk increases with age [8]. The cancer treatment also depends on the age of the patient. In patients under the age of 70, maximal surgical resection in combination with radiotherapy and adjuvant chemotherapy with temozolomide and carmustin is used. In the case of metastasis, alkylating chemotherapy with lomustine is used. Despite the complex treatment, recurrences are very common, with the tumor spreading to other areas of the brain. Metastases outside the brain are infrequent [9].

In turn, hepatocellular carcinoma (HCC) accounts for approximately 75% of primary liver tumors and is also the most common primary tumor of this organ. HCC is more common in men than in women worldwide, and in 2020, in this population, hepatocellular carcinoma was the second highest cause of death from cancer [10,11]. The prevalence of this type of cancer increases with age, with the peak incidence occurring at the age of 70 [11]. Among the risk factors, viral hepatitis and alcohol abuse must be highlighted, however, non-alcoholic fatty liver disease is rapidly becoming a dominant cause of HCC [12]. Systemic treatment is used in patients with lesions that do not qualify for surgical removal or in patients awaiting liver transplantation. Due to the lack of confirmed efficacy for conventional cytostatics, molecular-based drugs are used. The recommended first-line treatment for hepatocellular carcinoma in patients without contraindications should be bevacizumab with atezolizumab [13].

Another cancer characterized by higher prevalence occurring in men than in women is renal cell carcinoma (RCC). This is the most common type of urological cancer and represents more than 90% of all kidney cancers. Renal cancer occurs mostly in European and North American populations. The mortality rate is 30–40% and is higher compared to other urological cancers, such as bladder or prostate cancer. In addition to gender, other risk factors of RCC include obesity, hypertension, smoking, and chronic kidney disease. RCC is most common in people between the ages of 60 and 70, with the incidence decreasing after the age of 70. The prognosis of survival is affected by the stage at which the disease is diagnosed and the presence or absence of metastases to other organs. Approximately 20–30% of renal cell carcinoma cases are discovered when a patient has already metastasized, which significantly affects the subsequent prognosis. The treatment depends on the size of the tumor and the presence of metastases [14]. Systemic treatment includes immunotherapy, the usage of vascular endothelial growth factor (VEGF) receptor inhibitors (bevacizumab) and tyrosine kinase inhibitors (sunitinib and pazopanib), as well as mammalian target of rapamycin (mTOR) kinase inhibitors (everolimus and temsyrolimus) [15].

Hydrazide–hydrazones, on the basis of the latest literature findings and above-presented needs, constitute a promising class of organic compounds for the treatment of infections and in cancer therapy. Beside this, they are used in the synthesis of many heterocyclic systems and can be applied as ligands to obtain bioactive metal complexes [16,17,18,19,20,21,22,23,24,25]. Molecules with a hydrazide–hydrazone moiety in their structure are therapeutically promising new substances, in particular due to their antimicrobial and anticancer potential, and they should be highlighted in view of the increasing incidence of malignant neoplasms and resistant bacterial and fungal infections worldwide [6,10,22,23,24,25,26,27,28,29,30,31,32,33,34]. It is also worth underlining that nitrofurazone [35], furazolidone [36], and nitrofurantoin [37,38] are widely used chemotherapeutic medicines known to possess a hydrazide–hydrazone moiety.

Additionally, we would like to mention that different derivatives containing a hydrazide–hydrazone moiety were tested and proved to be cytotoxic against human cancer cell lines derived from lung cancer (NCI-H460 and A549), colon cancer (COLO 205 and HCT 15), central nervous system tumors (SF-268 and SH-SY-5Y), breast cancer (MCF-7 and MDA-MB-231), ovary cancer (SK-OV-3), cervical cancer (HeLa), gastric cancer (BGC-823), liver cancer (HepG2), renal cancer (769-P), and leukemia (Jurkat and BV-173) [26,27,28,29,30,31,32,33,34,39,40,41,42,43,44,45,46,47,48]. In addition, in our previous studies [31,32] as well as in research published by other scientific teams, hydrazide–hydrazones were found to be very active towards human hepatocellular carcinoma (HepG2) [46,47,48,49]. Taha et al. proved that their antiproliferative effect was stronger than that observed with the well-known and widely used cytostatic drug doxorubicin [46]. Moreover, El-Faham et al. demonstrated that newly synthesized hydrazide–hydrazones were selective towards HepG2 cells and inhibited their viability four times better than 5-fluorouracil [29].

The treatment of malignant tumors is still a challenge for modern medicine. However, every year, the amount of research on novel compounds increases, which gives hope for their future use and obtaining new drugs that will be efficacious and, above all, as safe as possible. The available cytostatic agents are often not selective towards cancer cells but also affect healthy cells, causing serious sides effects. Newly developed anticancer medicines should be highly specific and have as few adverse effects as possible, making therapy effective and comfortable for the patient.

Therefore, aside from establishing antimicrobial activity, the aim of our study was also an evaluation of the in vitro anticancer potential and in vivo toxicity of the selected synthesized hydrazide–hydrazones. The antimicrobial activity was assessed on the basis of assays performed according to EUCAST (European Committee for Antimicrobial Susceptibility Testing) and CLSI (Clinical and Laboratory Standards Institute) guidelines [50,51]. The antiproliferative experiments were conducted on selected human cancer cell lines, 769-P (renal adenocarcinoma), HepG2 (hepatocellular carcinoma), H1563 (lung adenocarcinoma), and LN-229 (glioblastoma), as well as on a human embryonic kidney cell line (HEK-293) as a reference line. The effects of the tested substances on cell viability were assessed using an MTT assay, which measures cell metabolic activity, and on the basis of results obtained, the inhibitory concentration (IC_50_) values were determined [52]. The in vivo toxicity assessment was performed in a zebrafish embryo model (*Danio rerio*) using the Fish Embryo Acute Toxicity Test (FET) (OECD No. 236), which involved the microscopic observation of lethal developmental abnormalities and determination of LC_50_ values based on these [53]. In addition, the occurrence of other non-lethal morphological malformations and the effects of the substances on heart action were measured.

The choice of 2,4-dihdroxybenzoic acid hydrazide as a starting compound for the synthesis was based on reports from the literature. According to the scientific literature, 2,4-dihydroxybenzoic acid with two hydroxyl groups bonded to the benzene ring in the *meta* position in relation to each other showed moderate antioxidant and low DPPH^•^ (1,1-diphenyl-2-picrylhydrazyl radical) and hydrogen peroxide scavenging activity [54]. Spiegel et al. also confirmed that 2,4-dihydroxybenzoic acid was much less efficient than other phenolic acids and exhibited significantly lower antioxidant activity [55]. 2,4-dihydroxybenzoic acid as phenolic compound was identified in wild mushrooms by Alves et al. The authors tested its antibacterial activity against a panel of Gram-positive and Gram-negative bacterial strains. On the basis of the conducted research, the authors underlined that among other isolated and tested phenolic acids, only 2,4-dihydroxybenzoic acid showed activity against Gram-negative bacteria (*Escherichia coli*, *Pasteurella multocida*, and *Neisseria gonorrhoeae* with MIC = 1.0 mg/mL). It also inhibited the growth of Gram-positive bacterial strains including the *Staphylococcus aureus* MRSA strain (MIC = 0.5 mg/mL) and *Enterococcus faecalis* (MIC = 1.0 mg/mL) [56]. The studies conducted by Kalinowska et al. revealed that 2,4-dihydroxybenzoic acid possessed the strongest antimicrobial properties towards *E. coli*, *P. aeruginosa*, *S. aureus*, *B. subtilis*, *S. enteritidis*, and *C. albicans* at a concentration of 2 mg/mL. 2,4-dihydroxybenzoic acid has been found to exhibit significant antimicrobial properties against various pathogens, including *Salmonella*, *E. coli*, and *L. monocytogenes*. Additionally, the effects of seven hydroxybenzoic acids, including 2,4-dihydroxybenzoic acid, on two types of human breast cancer cell lines (MCF-7 and MDA-MB-231) were investigated. The cytotoxicity determined by the IC_50_ parameter (the concentration of the sample that inhibited cell viability by 50% compared to control cells) for 2,4-dihydroxybenzoic acid was 4.77 mM towards the MDA-MB-231 cell line. Against the MCF-7 cell line, 2,4-dihydroxybenzoic acid was nontoxic [57].

On the basis of the above-mentioned facts in this research, we focused on the antimicrobial and antiproliferative activity of synthesized hydrazide–hydrazones of 2,4-dihydroxybenzoic acid with the aim of confirming their potential bioactivity and application in treatment.

## 2. Results

### 2.1. Chemistry

**Scheme 1:**
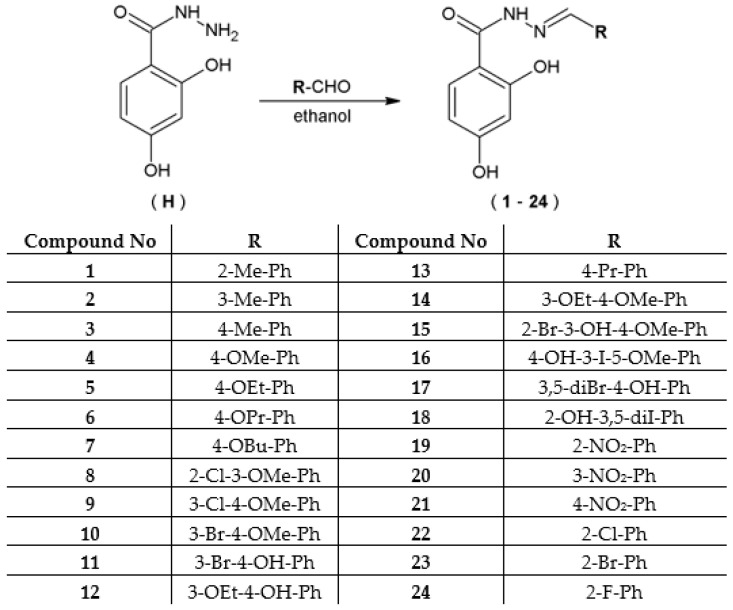
Synthesis of hydrazide–hydrazones of 2,4-dihydroxybenzoic acid.

In this research, we synthesized twenty-four derivatives of 2,4-dihydroxybenzoic acid with hydrazide–hydrazone moiety. The titled compounds were synthesized on the basis of a condensation reaction of 2,4-dihydroxybenzoic acid hydrazide (**H**) with appropriate aromatic aldehydes, according to the procedure which we described earlier [24,25,31,32]. The time required for the reaction was established experimentally (15–40 min) (Scheme 1).

The yield of the condensation reaction was in the range of 23–98%. The highest yield, 98%, was found for the preparation of 2,4-dihydroxy-*N*-[(4-methylphenyl)methylidene]benzohydrazide (**3**), *N*-[(4-butoxyphenyl)methylidene]-2,4-dihydroxybenzohydrazide (**7**), and *N*-[(3-bromo-4-hydroxyphenyl)methylidene]-2,4-dihydroxybenzohydrazide (**11**), whereas the lowest yield, 23%, was found for the synthesis of 2,4-dihydroxy-*N*-[(4-propylphenyl)methylidene]benzohydrazide (**13**). The melting points for the synthesized compounds were in the range of 197–263 °C. The highest melting temperature was measured for 2,4-dihydroxy-*N*-[(4-methoxypenyl)methylidene]benzohydrazide (**4**), 263 °C, and the lowest, 197 °C, was found for 2,4-dihydroxy-*N*-[(4-propylphenyl)methylidene]benzohydrazide (**13**). The yield of the reactions, the time of the reactions, and the melting points of the synthesized compounds are summarized in Table 1.

In order to confirm the correctness of the condensation reaction and the chemical structure of the synthesized hydrazide–hydrazones of 2,4-dihydroxybenzoic acid (**1**–**24**), we performed an analysis of ^1^H NMR and ^13^C NMR spectra. The ^1^H NMR and ^13^C NMR spectra of the selected compounds are presented in the Supplementary Materials (Figures S1–S10).

### 2.2. Microbiology—Antimicrobial Activity

The antimicrobial activity of the newly synthesized compounds **1**–**24** was tested towards reference Gram-positive and Gram-negative bacteria. Moreover, the antifungal effect against yeasts belonging to *Candida* spp. was investigated.

As presented in Table 2, Table 3 and Table 4, some of these compounds showed antimicrobial effects. All reference Gram-positive bacteria were sensitive to compounds **2**, **9**, **10**, **11**, **17**, **18**, **19**, and **21**. The minimal inhibitory concentrations of these substances, which influenced the growth of the reference *Staphylococcus* spp., *Enterococcus faecalis*, *Micrococcus luteus*, and *Bacillus* spp., were in a wide range (MICs from 0.48 to 1000 µg/mL; minimal bactericidal concentrations (MBCs) from 1.95 to >1000 µg/mL). Among them, compound **18** showed very strong antibacterial activity with an MIC in the range of 0.48–7.81 µg/mL, MBC = 1.95–31.25 µg/mL, and a bactericidal (MBC/MIC = 2–4) or bacteriostatic effect (MBC/MIC = 8) towards these microorganisms. Another compound **9**, also indicated a high level of activity, with a strong (MIC = 15.62 µg/mL) or good (MIC = 31.25–125 µg/mL) bactericidal effect (MBC/MIC = 1–4) against the Gram-positive bacteria. The remaining hydrazide–hydrazones **2**, **10**, **11**, and **17** showed slightly lower activity, with MICs ranging from 15.62 to 1000 µg/mL and MBCs ranging from 250 to >1000 µg/mL. Their activity was mostly good or moderate. In the case of substance **13**, the antibacterial effect was mild, with MIC = 1000 µg/mL and MBC > 1000 µg/mL. The remaining compounds **1**, **5**–**7**, and **14**–**16** indicated a much weaker effect or none at all. *Micrococcus luteus* was the most sensitive to them (Table 2 and Table 3).

Substances **2**, **13**, **14**, **17**, and **18** showed only mild activity against some of the Gram-negative bacteria (MIC = 500–1000 µg/mL and MBC > 1000 µg/mL) or had no activity. As in the case of the Gram-positive microorganisms, hydrazide–hydrazone **18** was also the most active among these compounds against reference strains of *Bordetella bronchiseptica*, *Escherichia coli*, *Klebsiella pneumoniae*, *Proteus mirabilis*, *Salmonella typhimurium*, and *Pseudomonas aeruginosa* (Table 2 and Table 3).

The sensitivity of the fungi belonging to reference *Candida* spp. to the tested substances was low. Compounds **2**, **5**–**7**, and **13**–**18** showed moderate or mild effects (with MIC = 250–1000 µg/mL and MFC = 500–>1000 µg/mL) or no antifungal bioactivity (MIC = >1000 µg/mL). *C. albicans* strains were the most sensitive to them. In turn, compound **18** indicated some inhibitory effect towards all yeast strains (MIC = 500–1000 µg/mL) (Table 2 and Table 3).

Among compounds **19**–**24**, as presented in Table 4, the widest spectrum of activity towards all microorganisms was exhibited by compound **19**. Its activity was moderate or mild towards Gram-positive (MIC = 250–1000 µg/mL) and Gram-negative bacteria (MIC = 500–1000 µg/mL). Moreover, this compound influenced the growth of *Candida* spp. strains with MICs between 500 and 1000 µg/mL and with mild effects. Gram-positive bacteria were also minimally sensitive to compound **21** (MIC = 500–1000 µg/mL) (Table 4).

Compounds **22** and **23** inhibited the growth of reference *S. epidermidis*, as well as **24** *Enterococcus faecalis*, *Micrococcus luteus*, and *Bacillus* spp. strains. Gram-negative rods from *Enterobacterales* and non-fermenting bacteria (*P. aeruginosa*) were insensitive to the studied compounds **19**–**24**, except for hydrazide–hydrazone **19**. In the case of the fungi belonging to the reference *Candida* spp., the activity of other compounds (except substance **19**) was similar (with MIC = 500–1000 µg/mL and MFC > 1000 µg/mL), or they were inactive. *C. glabrata* was susceptible only to compound **19** (Table 4).

### 2.3. In Vitro Assessment of the Antiproliferative Potential of the Tested Hydrazide–Hydrazones

The highest antiproliferative activity results of hydrazide–hydrazones of 5-bromo-2-iodobenzoic acid with chloro and nitro substituents at the phenyl ring published previously [32] encourage us to choose the compounds numbered **19**–**24** to confirm if the above-mentioned substituents are crucial to increase the antiproliferative potential of hydrazide–hydrazones in the case of 2,4-dihydroxybenzoic acid derivatives.

According to the IC_50_ values calculated for all tested compounds (**19**–**24**), their high selectivity was proved. The tested hydrazide–hydrazones were more cytotoxic towards lung, renal, and liver cancer cells as well as glioblastoma than the normal cell line (HEK-293) (Table 5). The highest antiproliferative potential was shown for *N*-[(4-nitrophenyl)methylidene]-2,4-dihydroxybenzhydrazide (**21**). In the case of this substance, LN-229 (glioblastoma) was found to be the most sensitive cancer cell line, and the IC_50_ value was estimated at 0.77 µM. The above-mentioned compound inhibited the viability of three of the four used cancer cell lines, with very low IC_50_ values estimated at 7.81 µM for hepatocellular carcinoma (HepG2) and 12.39 µM for renal adenocarcinoma (769-P) (Table 5). Promising antiproliferative properties were also exhibited by the derivative which was substituted with a nitro group at position two in the phenyl ring. The *N*-[(2-nitrophenyl)methylidene]-2,4-dihydroxybenzhydrazide (**19**) was the most cytotoxic towards 769-P cells and similarly active against the H1563 line (lung cancer), with IC_50_ values estimated at 45.42 µM and 65.67 µM, respectively. On the contrary, the lowest antiproliferative activity of this compound was noted for LN-229 (IC_50_ = 130.17 µM) (Table 5). In turn, *N*-[(3-nitrophenyl)methylidene]-2,4-dihydroxybenzhydrazide (**20**) demonstrated anticancer potential only against lung adenocarcinoma (H1563), with an IC_50_ value estimated at 70.94 µM (Table 5).

In turn, replacing the nitro group with a halogen substituent led to a significant decrease in the antiproliferative potential of newly developed molecules (Table 5). They proved to inhibit the viability of glioblastoma (LN-229) and lung adenocarcinoma (H1563) in particular. However, the calculated inhibitory concentrations were higher and ranged from 100 to 200 µM (Table 5). Among these substances, the most promising seemed to be *N*-[(2-bromophenyl)methylidene]-2,4-dihydroxybenzhydrazide (**23**), with IC_50_ values estimated at 101.14 µM for H1563 and 156.77 µM for LN-229 (Table 5).

### 2.4. In Vivo Toxicity Assessment of Hydrazide–Hydrazones in the Zebrafish Model

**Figure 1:**
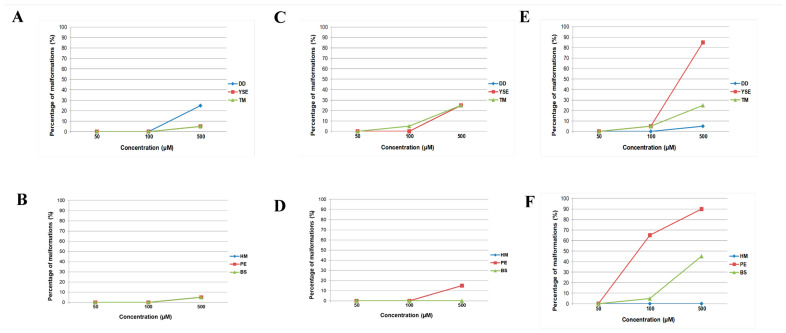
The percentage of developmental malformations observed in zebrafish larvae at the age of 96 hpf which were exposed to tested hydrazide–hydrazones ((**A**,**B**)—compound **19**; (**C**,**D**)—compound **20**; (**E**,**F**)—compound **24**). Malformations: BS—bent spine, DD—developmental delay, HM—head malformation, PE—pericardium edema, TM—tail malformation, YSE—yolk sac edema.

**Figure 2:**
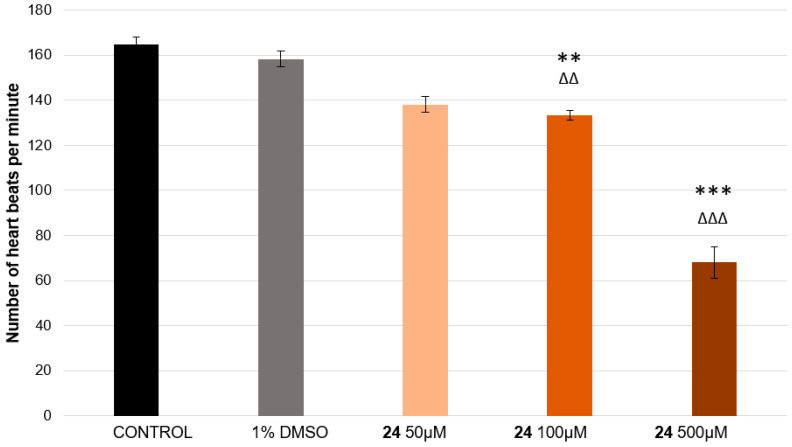
The average heart rate of zebrafish larvae after 96-hour exposure to control solutions (negative control—E3; solvent control—1% DMSO) and tested substance solutions (**24**). The results are presented as mean ± SEM. Statistical analysis was performed using parametric one-way ANOVA, followed by Bonferroni’s post hoc test, with a significance level of *p* < 0.05. *** *p* < 0.001 and ** *p* < 0.01 compared to E3; ∆∆∆ *p* < 0.001 and ∆∆ *p* < 0.01 compared to 1% DMSO.

The results of the in vivo studies presented in this paper indicated that *N*-[(2-nitrophenyl)methylidene]-2,4-dihydroxybenzhydrazide (**19**) and *N*-[(3-nitrophenyl)methylidene]-2,4-dihydroxybenzhydrazide (**20**) were actually nontoxic for zebrafish embryos and larvae. As shown in Table 6, the values of half-maximal lethal concentration (LC_50_) were estimated at 1.88 × 10^5^ µM (5.68 × 10^4^ mg/L) and 3.48 × 10^4^ µM (1.05 × 10^4^ mg/L), respectively. In addition, the percentage of non-lethal morphological abnormalities did not exceed 25% (Figure 1). Based on the LC_50_ value, *N*-[(2-fluorophenyl)methylidene]-2,4-dihydroxybenzhydrazide (**24**) also seemed to be nontoxic in the zebrafish model. However, the analysis of morphological and physiological defects in the exposed embryos and larvae demonstrated that at concentrations of 100 µM (27.42 mg/L) and 500 µM (137.12 mg/L), high percentages of pericardial edema was observed, 65% and 85%, respectively (Figure 1). The percentage of pericardial edema is plausibly associated with bradycardia noted in 96 hpf larvae exposed to higher concentrations of *N*-[(2-fluorophenyl)methylidene]-2,4-dihydroxybenzhydrazide (**24**) (Figure 1 and Figure 2). When zebrafish embryos were exposed to *N*-[(2-fluorophenyl)methylidene]-2,4-dihydroxybenzhydrazide (**24**) at a concentration of 50 µM (13.71 mg/L), no changes (*p* > 0.05) in the heart rate were observed after 96 h compared to both controls, i.e., negative (E3) and solvent (1% DMSO) (Figure 2), while at a concentration of 100 µM (27.42 mg/L), a statistically significant (F[2.21] = 63.793; *p* < 0.01) decrease in heartbeat (bradycardia) was already observed, and for 500 µM (137.12 mg/L), the reduction was very statistically significant (F[2.21] = 63.793; *p* < 0.001) when compared to the negative and solvent controls (Figure 2).

**Figure 3:**
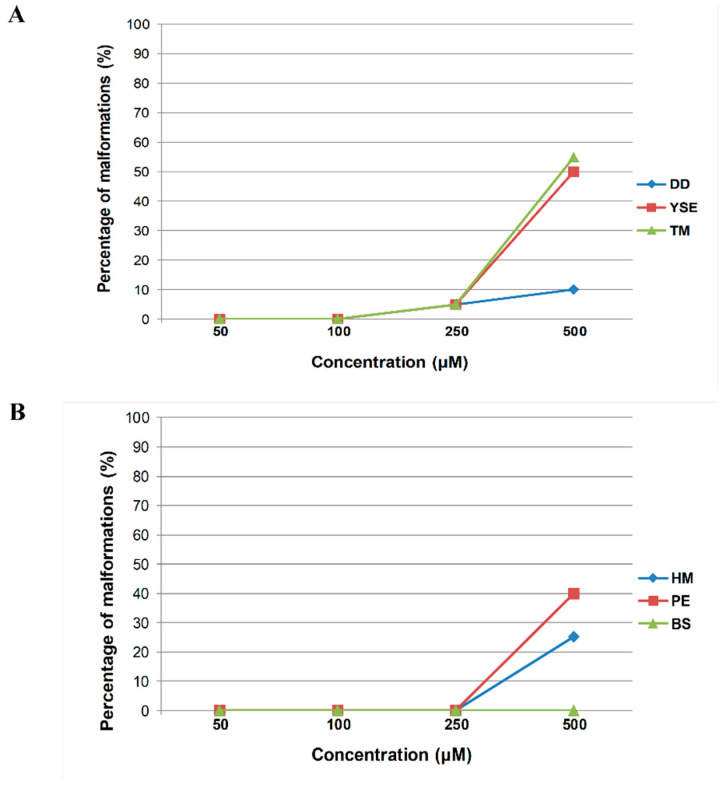
The percentage of developmental malformations observed in 96 hpf zebrafish larvae exposed to tested hydrazide-hydrazone ((**A**,**B**)—compound **21**). Malformations: BS—bent spine, DD—developmental delay, HM—head malformation, PE—pericardium edema, TM—tail malformation, YSE—yolk sac edema.

In turn, assessed in the zebrafish model, *N*-[(4-nitrophenyl)methylidene]-2,4-dihydroxybenzhydrazide (**21**) was characterized by moderate toxicity, with an LC_50_ value calculated at 370 µM (110 mg/L) (Table 6). In addition, as seen in Figure 3, the percentage of morphological abnormalities already significantly increased at a concentration of 500 µM (150.62 mg/L) and ranged from 10% for the occurrence of development delay to 55% for tail malformation (Table 6).

**Figure 4:**
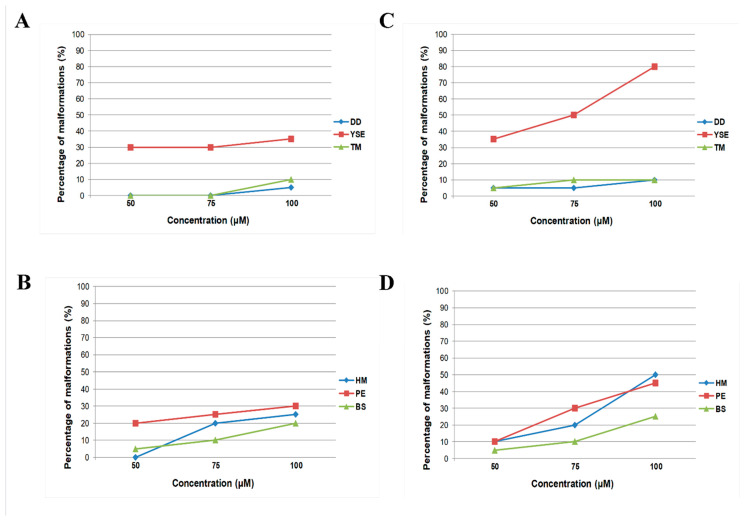
The percentage of developmental malformations observed in zebrafish larvae at the age of 96 hpf which were exposed to tested hydrazide–hydrazones ((**A**,**B**)—compound **22**; (**C**,**D**)—compound **23**). Malformations: BS—bent spine, DD—developmental delay, HM—head malformation, PE—pericardium edema, TM—tail malformation, YSE—yolk sac edema.

In contrast, *N*-[(2-chlorophenyl)methylidene]-2,4-dihydroxybenzhydrazide (**22**) and *N*-[(2-bromophenyl)methylidene]-2,4-dihydroxybenzhydrazide (**23**) were shown to be quite toxic to zebrafish embryos. This was confirmed by the calculated LC_50_ values for individual compounds, i.e., 56.56 µM (16.44 mg/L) for **22** and 85.62 µM (30.41 mg/L) for **23** (Table 6), as well as the percentage of occurrence of physiological and morphological abnormalities (Figure 4). Sub-lethal deformities were already observed in *Danio rerio* embryos and larvae exposed at a concentration of 50 µM. As shown in Figure 4, yolk sac edema was the most common morphological deformity noted at the highest level of 80% in the group of embryos and larvae exposed to compound **23** at the concentration of 100 µM (35.52 mg/L) (Table 6).

## 3. Discussion

### 3.1. Chemistry

The condensation reaction of hydrazide of 2,4-dihydroxybenzoic acid enabled the synthesis of twenty-four hydrazide–hydrazones (**1**–**24**). On the basis of the yields of the performed reactions, it can be seen that the condensation reactions with aldehydes which possessed alkyl, hydroxyl, or alkoxyl groups were characterized by the highest yields.

According to the scientific literature, to confirm the chemical structure of hydrazide–hydrazones, spectroscopic methods (^1^H NMR and ^13^C NMR) may be applied [17,18]. In the ^1^H NMR spectra, researchers should look for the singlet signal for the proton of =CH group at δ 8–9 ppm and another singlet signal for the proton of the NH group in the range of δ 10–13 ppm. According to the literature, in the ^13^C NMR spectra, hydrazide–hydrazones show the signal of the carbon atom of the =CH group at around δ 145–160 ppm and that of the carbon atom of the carbonyl group (C=O) in the range of δ 160–170 ppm.

An analysis of the spectra of hydrazide–hydrazones **1**–**24** obtained in this research revealed that the analyzed substances possessed characteristic signals both in the ^1^H NMR and ^13^C NMR spectra. In the ^1^H NMR of the synthesized substances (**1**–**24**), we found two characteristic signals as singlets, one for the proton of the =CH group (δ 7.91–8.86 ppm) and the second one for the proton of the NH group (δ 11.55–12.05 ppm). On the other hand, the analysis of the ^13^C NMR spectra revealed signals for the carbon atom of the =CH group (δ 141.12–149.35 ppm) and for the carbon atom of the carbonyl group (δ 165.44–169.12 ppm).

The chemical shift values for the analyzed fragments of the synthesized hydrazide–hydrazones were in comparable ranges, which proved the proper synthesis and chemical structure of the obtained substances. Additional signals for other aliphatic and aromatic groups of the analyzed compounds were found at optimal and expected values of chemical shift (δ).

### 3.2. Microbiology—Antimicrobial Activity

According to a literature survey and on the basis of our previously performed research and published articles, hydrazide–hydrazones of carboxylic acids possess significant antimicrobial activity [22,23,24,25,31,32]. On the basis of research conducted by our group previously, it can be concluded that substituents, especially substituents with halogen and methoxy groups in the phenyl ring, presented next to the nitrogen atom of hydrazide–hydrazones influence the antimicrobial activity the most [24,25,31,32].

In this study, the situation was similar: the highest activity against selected Gram-positive bacterial strains among all the synthesized compounds was shown by hydrazide–hydrazone **18** with a 2-hydroxy-3,5-diiodophenyl substituent. It is especially worth mentioning that the activity of this compound against *S. aureus* ATCC 43400 MRSA strain was two times higher (MIC = 3.91 μg/mL) than the activity of nitrofurantoin (MIC = 7.81 μg/mL). The same compound possessed an MIC value (MIC = 0.98 μg/mL) towards *S. epidermindis* ATCC 12228 (MIC nitrofurantoin = 3.91 μg/mL) that was four times lower. Against *M. luteus* ATCC 10240, this hydrazide–hydrazone (MIC = 0.48 μg/mL) displayed activity that was 130-fold higher than that of nitrofurantoin (MIC = 62.5 μg/mL) and two times higher than that of ciprofloxacin and cefuroxime (MIC = 0.98 μg/mL). The MIC value for this compound towards *B. subtilis* ATCC 6633 (MIC = 1.95 μg/mL) was 2 times lower than that of nitrofurantoin (MIC = 3.91 μg/mL), 8 times lower than that of cefuroxime (MIC = 15.62 μg/mL), and 32 times lower than that of ampicillin (MIC = 62.5 μg/mL). Towards another *Bacillus* strain, *B. cereus* ATCC 10876, its activity (MIC = 3.91 μg/mL) was two times higher than that of nitrofurantoin (MIC = 7.81 μg/mL) and eight times higher than that of cefuroxime (MIC = 31.25 μg/mL). Additionally, its antibacterial effect against Gram-positive bacterial strains was bactericidal (Table 3).

Among the other compounds which possessed significant activity, we would like to underline the activity of hydrazide–hydrazones **2** and **9**. The antibacterial activity of hydrazide–hydrazone **2**, which was substituted with a 3-methylphenyl substituent, was important, especially towards *M. luteus* ATCC 10240. The MIC value for this compound against this strain (MIC = 31.25 μg/mL) was two times lower than that of nitrofurantoin (MIC = 62.5 μg/mL). Towards *B. subtilis* ATCC 6633, this substance displayed activity equal to the activity of ampicillin (MIC = 62.5 μg/mL). Compound **9** substituted with 3-chloro-4-methoxyphenyl displayed the highest activity against *B. cereus* ATCC 10876. Towards this strain, its activity (MIC = 15.62 μg/mL) was two times higher than that of cefuroxime (MIC = 31.25 μg/mL) (Table 2).

### 3.3. Cytotoxicity

Cancer is a serious public health problem in all modern societies worldwide [3,4,5,6,10]. There is no doubt that in addition to existing treatment approaches, promising new treatment methods must be developed. Therefore, the activities of scientists who design, synthesize, and evaluate the safety of new chemical substances in the search for new, safe, and effective anticancer drugs are essential. Research conducted by various scientific teams, including the team of scientists who authored this research, proves that among other therapeutic properties, hydrazide–hydrazones also show antiproliferative activity [22,23,24,25,26,27,28,29,30,31,32,33,34].

In this study, newly synthesized hydrazide–hydrazones (*N*-[(2-nitrophenyl)methylidene]-2,4-dihydroxybenzhydrazide (**19**), *N*-[(3-nitrophenyl)methylidene]-2,4-dihydroxybenzhydrazide (**20**), *N*-[(4-nitrophenyl)methylidene]-2,4-dihydroxybenzhydrazide (**21**), *N*-[(2-chlorophenyl)methylidene]-2,4-dihydroxybenzhydrazide (**22**), *N*-[(2-bromophenyl)methylidene]-2,4-dihydroxybenzhydrazide (**23**), and *N*-[(2-fluorophenyl)methylidene]-2,4-dihydroxybenzhydrazide (**24**)) were subjected to in vitro antiproliferative studies as well as in vivo toxicity research. Human cancer cell lines derived from renal adenocarcinoma (769-P), hepatocellular carcinoma (HepG2), lung adenocarcinoma (H1563), and glioblastoma (LN-229), but also a human embryonic kidney cell line (HEK-293) as a reference line, were selected for the experiments. It must be highlighted that lung carcinoma is still the most commonly diagnosed cancer in men and women worldwide, and adenocarcinoma represents 40% of all diagnoses, even being found in patients who have never smoked [4,7]. However, it is glioblastoma that has the worst prognosis as a fast-growing and aggressive brain tumor. This is the most common malignant brain tumor, as well as other central nervous system tumors, accounting for 47.7% of all cases [8]. Renal cell carcinoma is another cancer with a poor prognosis because up to one in three patients is metastatic at the early stage of diagnosis. It is the most lethal urogenital cancer, with a mortality rate of 30–40% [14]. In turn, hepatocellular carcinoma is the second leading cause of cancer-related deaths in men and represents a major global healthcare challenge [10,11,12].

Among the hydrazide–hydrazones tested (**19**–**24**), *N*-[(4-nitrophenyl)methylidene]-2,4-dihydroxybenzhydrazide (**21**) showed the highest antiproliferative potential against most cancer lines. The IC_50_ value against the line LN-229 was found to be the most promising and equaled 0.77 µM. The cytotoxicity towards 769-P and HepG2 cells were also very significant, with IC_50_ values of 12.39 µM and 7.81 µM, respectively. At the same time, the selectivity of this compound was very high because substance **21** had almost no cytotoxic effect on the physiological cell line. Similarly, the remaining molecules were found to be selective against cancer cells, showing weak or practically no cytotoxicity towards the physiological HEK-293 line. In the case of LN-229, the antiproliferative potential of the other hydrazide–hydrazones tested was significantly weaker, and the IC_50_ ranged from 110.53 µM for *N*-[(2-chlorophenyl)methylidene]-2,4-dihydroxybenzhydrazide (**22**) to 199.32 µM for *N*-[(2-fluorophenyl)methylidene]-2,4-dihydroxybenzhydrazide (**24**). The lowest IC_50_ value applying to lung adenocarcinoma (H1563) was 65.57 µM and was estimated for *N*-[(2-nitrophenyl)methylidene]-2,4-dihydroxybenzhydrazide (**19**), but a similar value (70.94 µM) was found for *N*-[(3-nitrophenyl)methylidene]-2,4-dihydroxybenzhydrazide (**20**). The IC_50_ values obtained for the cancer cell lines tested in this study, such as 769-P and HepG2, were similar to those estimated for the other hydrazide–hydrazones with the phenyl ring substituted with a nitro group at position 2 or 4, which were tested in our previous study [32]. These facts proved that a 4-nitrophenyl moiety is a desirable structure in hydrazide–hydrazones to obtain promising and varied antiproliferative activity in human cancer cell lines. Other scientific teams confirmed the anticancer potential of hydrazide–hydrazones, in particular in a hepatocellular carcinoma line (HepG2) [25,26,27]. Taha et al. showed that morpholine hydrazones inhibited the proliferation of HepG2 cells, and one of the tested compounds showed a particularly beneficial cytotoxic effect—its IC_50_ was estimated at 6.31 μM, compared to the standard doxorubicin (6.00 μM) and to the compound **21** presented in this study (7.81 μM) [46]. In turn, Nasr and colleagues tested new hydrazide–hydrazones obtained from coumarin derivatives for cytotoxic activity on HepG2 cells, and similarly for one of them, the IC_50_ was 4.89 μM and was lower than for doxorubicin [58]. The Krátký research team examined iodinated 1,2-diacylhydrazines and benzohydrazide–hydrazones and proved that most of the tested compounds had a cytotoxic effect on HepG2 cells, and the IC_50_ values for four of them were lower than for the standards of doxorubicin and tamoxifen [59].

Introducing to the structure of hydrazide–hydrazone a phenyl ring substituted with a halogen atom (chlorine, bromine, or fluorine) at position 2 significantly decreased the antiproliferative activity of the tested compounds in cancer cell lines used in the study. On the contrary, in our previous studies, the hydrazide–hydrazones with a 2-chlorophenyl ring inhibited the viability of 769-P cells several times more strongly [31,32]. In the presented research, the most active against human cancer line tested was hydrazide–hydrazone with a 2-bromophenyl moiety (**23**). The IC_50_ values for this compound were estimated at 101.14 µM (H1563) and 156.77 µM (LN-229). In addition, *N*-[(2-bromophenyl)methylidene]-2,4-dihydroxybenzhydrazide (**23**) practically had no impact on the viability of normal cells.

The search for new medicines is a complex and long-term process. Assessment of the potential toxicity of novel molecules as drug candidates is a crucial phase in preclinical studies, and animal models are widely used due to the fact that in vivo methods are the only ones acceptable to institutions for the safety assessment of compounds. The results of in silico and in vitro tests are still only an additional source of knowledge. In order to minimize the number of animals and their suffering, the 3R method is always considered. The premise of this principle is, among others, to refine the research to reduce the suffering and pain of the animals. Additionally, laboratory animals should have a small size, short life cycle, rapid growth, and high fertility, and more importantly in the case of the development of new drugs, it should be similar to humans. One of the models that meets these criteria is the zebrafish model. The high similarity to the human genome makes *Danio rerio* a good model organism for conducting studies in the area of toxicology and pharmacology [49,60].

The compound that was shown to be the most active against human cancer cells tested in this study, i.e., *N*-[(4-nitrophenyl)methylidene]-2,4-dihydroxybenzhydrazide (**21**), exhibited moderate toxicity in the zebrafish model based on the LC_50_ value estimated at 370 µM and the percentage of developmental abnormalities. In turn, *N*-[(2-nitrophenyl)methylidene]-2,4-dihydroxybenzhydrazide (**19**) presented promising antiproliferative potential, in particular in 769-P and H1563 cells, showing negligible toxicity against zebrafish embryos and larvae. Our results are confirmed by previous studies that proved that a phenyl ring in the structure of the molecule that is substituted with a nitro group at position 4 is more toxic to zebrafish than that substituted at position 2 [61,62]. However, in our in vivo model, the highest toxicity against zebrafish embryos occurred when the phenyl ring was substituted with chlorine or bromine at position 2, whereas *N*-[(2-fluorophenyl)methylidene]-2,4-dihydroxybenzhydrazide (**24**) seemed to be nontoxic in the zebrafish model when based only on the LC_50_ value estimated at 4.24 × 10^3^ µM. However, the percentage of morphological deformities in the exposed embryos and larvae was demonstrated to be high in the case of pericardial edema that was associated with bradycardia. Melong and others studied the embryo toxicity of enzalutamide, a cytostatic containing in its structure a fluorine atom at position 2 of the benzene ring, in *Danio rerio* [63]. Similarly, they showed that the mortality rate of zebrafish larvae increased in a dose-dependent manner, and the bradycardia significantly increased, which was comparable to the effect caused by the positive control with terfenadine used as a cardiotoxicity standard. In addition, Kovács et al. proved that the exposure of zebrafish to another cytostatic, 5-fluorouracil, led to histopathological changes in the liver and kidney, along with a genotoxic effect (DNA damage) [64]. It may be concluded that the presence of a fluorine atom in the structure of the molecule can be responsible for higher toxicity and a potential cardiotoxic effect.

Despite many therapies being available, including conventional cytostatics, immunotherapy, monoclonal antibodies, and gene therapies, a large number of cancers are still not completely curable. Treatment failures and high mortality are the reasons why new drugs with anticancer properties are constantly being developed [3,4,5,6,10]. Unfortunately, side effects are an integral part associated with the treatment process with anticancer drugs. The occurrence of unwanted effects often requires the use of further symptomatic medicines, which can additionally affect the patient’s condition [7,8,9,13,14,15]. Drugs with as few side effects as possible that are selective towards rapidly dividing cells are highly desirable in medicine. The results presented in this work proved that newly developed hydrazide–hydrazones, in particular with a nitrophenyl moiety, show very promising and varied anticancer potential. *N*-[(4-nitrophenyl)methylidene]-2,4-dihydroxybenzhydrazide (**21**) showed the highest activity against human cancer line tested and its strong cytotoxic effect against glioblastoma must be highlighted. In turn, *N*-[(2-nitrophenyl)methylidene]-2,4-dihydroxybenzhydrazide (**19**) was found to be the most active against lung adenocarcinoma. Simultaneously, these compounds exhibited negligible or moderate toxicity in the in vivo zebrafish model. However, further studies should be performed to obtain a complete picture of the therapeutic potential and mechanisms of action of the tested compounds.

## 4. Materials and Methods

### 4.1. Chemistry

All reagents and solvents used in this research were purchased from Sigma-Aldrich (Munich, Germany) or Merck Co. (Darmstadt, Germany) and used as delivered by manufacturer. Thin layer chromatography (TLC) on aluminum plates covered with silica gel (aluminum oxide 60 F-254, Merck Co., Rahway, NJ, USA) was used to check the purity of the obtained compounds and to monitor the progress of the reactions. Chloroform-ethanol mixture 10:1 (*v*/*v*) ratio was used as the mobile phase. The spots were detected by irradiation with UV light at a λ = 254 nm. The ^1^H NMR and ^13^C NMR spectra were recorded on the Bruker Avance 300 and 600 apparatus (Bruker BioSpin GmbH, Ettlingen, Germany). The melting points of the obtained compounds were measured with a Fisher-Johns apparatus (Fisher Scientific, Dreieich, Germany), and presented without any correction. The elemental analysis was determined by a Perkin Elmer 2400 series II CHNS/O analyzer (Perkin Elmer, Waltham, MA, USA), and the results were within ± 0.4% of the theoretical value.

### 4.2. Procedure of the Synthesis of Hydrazide–Hydrazones of 2,4-Dihydroxybenzoic Acid

Target compounds were synthesized on the basis of the procedure reported earlier by Popiołek et al. [24,25,31,32]. First, 0.001 mole of the hydrazide of 2,4-dihydroxybenzoic acid (**H**) was dissolved in ethanol (96%, 5 mL) by heating under reflux. Then, 0.0011 mole of the appropriate aromatic aldehyde was added, and the mixture was heated under reflux until a precipitate appeared. The time of heating was 15 min (compound: **2**), 16 min (compounds: **1** and **3**), 17 min (compound: **5**), 18 min (compound: **4**), 20 min (compound: **6**), 23 min (compound: **7**), 26 min (compound: **8**), 29 min (compound: **11**), 30 min (compound: **9**), 31 min (compounds: **10** and **14**), 33 min (compounds: **15** and **19**), 36 min (compounds: **12**, **20**, and **21**), 37 min (compounds: **17** and **22**), 39 min (compound: **16**), and 40 min (compounds: **13**, **18**, **23**, and **24**) (Table 1). Subsequently, the mixture was cooled at room temperature, and the formed precipitate was filtered off under reduced pressure, dried, and subjected to re-crystallization in ethanol (96%) (Scheme 1).

#### Detailed Physico-Chemical Properties of Hydrazide–Hydrazones of 2,4-Dihydroxybenzoic Acid (**1**–**24**)

2,4-dihydroxy-*N*-[(2-methylphenyl)methylidene]benzohydrazide (**1**)

CAS: 405220-38-4; brown powder; yield: 83%; M.p.: 250 °C; ^1^H NMR (300 MHz, DMSO-*d_6_*): 2.45 (s, 3H, CH_3_), 6.32–6.40 (m, 2H, ArH), 7.24–7.32 (m, 3H, ArH), 7.80–7.85 (m, 2H, ArH), 8.72 (s, 1H, =CH), 10.24 (s, 1H, OH), 11.69 (s, 1H, NH), 12.43 (s, 1H, OH); ^13^C NMR (75 MHz, DMSO-*d_6_*): 19.54 (CH_3_), 103.36, 106.49, 107.85, 126.41, 126.65, 129.93, 130.29, 131.35, 132.66, 137.44 (10C_ar_), 147.15 (=CH), 163.01, 163.22 (2C_ar_), 166.05 (C=O).

2,4-dihydroxy-*N*-[(3-methylphenyl)methylidene]benzohydrazide (**2**)

CAS: 2487399-14-2; light yellow powder; yield: 96%; M.p.: 205 °C; ^1^H NMR (300 MHz, DMSO-*d_6_*): 3.35 (s, 3H, CH_3_), 6.31–6.39 (m, 2H, ArH), 7.24–7.31 (m, 1H, ArH), 7.32–7.37 (t, 1H, ArH, *J* = 6 Hz, *J* = 9 Hz), 7.49–7.56 (m, 2H, ArH), 7.79–7.82 (d, 1H, ArH, *J* = 9 Hz), 8.39 (s, 1H, =CH), 10.22 (s, 1H, OH), 11.68 (s, 1H, NH), 12.36 (s, 1H, OH); ^13^C NMR (75 MHz, DMSO-*d_6_*): 21.36 (CH_3_), 103.32, 106.66, 107.87, 125.06, 127.84, 129.21, 130.17, 131.33, 134.64, 138.55 (10C_ar_), 148.48 (=CH), 162.76, 163.17 (2C_ar_), 165.91 (C=O).

2,4-dihydroxy-*N*-[(4-methylphenyl)methylidene]benzohydrazide (**3**)

CAS: 364050-94-2; light brown powder; yield: 98%; M.p.: 248 °C; ^1^H NMR (600 MHz, DMSO-*d_6_*): 3.37 (s, 3H, CH_3_), 6.49–6.50 (m, 2H, ArH), 7.29–7.31 (d, 2H, ArH, *J* = 12 Hz), 7.69–7.71 (d, 2H, ArH, *J* = 12 Hz), 7.83–7.85 (m, 2H, ArH), 8.69 (s, 1H, =CH), 10.37 (s, 1H, OH), 11.73 (s, 1H, NH), 12.33 (s, 1H, OH); ^13^C NMR (150 MHz, DMSO-*d_6_*): 19.67 (CH_3_), 103.38, 107.43, 108.85, 127.35, 129.15, 129.93, 130.84, 131.90, 138.51 (10C_ar_), 149.35 (=CH), 161.57, 163.21 (2C_ar_), 167.01 (C=O).

2,4-dihydroxy-*N*-[(4-methoxyphenyl)methylidene]benzohydrazide (**4**)

The NIR-FT Raman and FT-IR spectra of this molecule have been recorded and analyzed earlier [65]. CAS: 405220-69-1; light yellow powder; yield: 95%; M.p.: 263 °C; ^1^H NMR (300 MHz, DMSO-*d_6_*): 3.80 (s, 3H, OCH_3_), 6.30–6.38 (m, 2H, ArH), 7.01–7.03 (d, 2H, ArH, *J* = 6 Hz), 7.66–7.69 (d, 2H, ArH, *J* = 9 Hz), 7.78–7.81 (d, 1H, ArH, *J* = 9 Hz), 8.37 (s, 1H, =CH), 10.21 (s, 1H, OH), 11.58 (s, 1H, NH), 12.43 (s, 1H, OH); ^13^C NMR (75 MHz, DMSO-*d_6_*): 55.75 (OCH_3_), 103.31, 106.65, 107.81, 114.82, 127.21, 129.23, 130.00 (9C_ar_), 148.39 (=CH), 161.36, 162.78, 163.07 (3C_ar_), 165.80 (C=O).

*N*-[(4-ethoxyphenyl)methylidene]-2,4-dihydroxybenzohydrazide (**5**)

CAS: 769143-61-5; brownish powder; yield: 94%; M.p.: 248 °C; ^1^H NMR (300 MHz, DMSO-*d_6_*): 1.31–1.36 (t, 3H, CH_3_, *J* = 9 Hz, *J* = 6 Hz), 4.04–4.11 (q, 2H, CH_2_, *J* = 6 Hz), 6.98–7.01 (d, 2H, ArH, *J* = 9 Hz), 7.64–7.67 (d, 2H, ArH, *J* = 9 Hz), 7.78–7.81 (d, 1H, ArH, *J* = 9 Hz), 8.37 (s, 1H, =CH), 10.20 (s, 1H, OH), 11.57 (s, 1H, NH), 12.43 (s, 1H, OH); ^13^C NMR (75 MHz, DMSO-*d_6_*): 15.04 (CH_3_), 63.73 (CH_2_), 103.31, 106.63, 107.80, 115.22, 127.06, 129.24, 129.98 (9C_ar_), 148.32 (=CH), 160.66, 162.79, 163.07 (3C_ar_), 165.81 (C=O).

2,4-dihydroxy-*N*-[(4-propoxyphenyl)methylidene]benzohydrazide (**6**)

CAS: 2488348-07-6; light yellow powder; yield: 95%; M.p.: 251 °C; ^1^H NMR (300 MHz, DMSO-*d_6_*): 0.95–1.00 (t, 3H, CH_3_, *J* = 9 Hz, *J* = 6 Hz), 1.68–1.80 (m, 2H, CH_2_), 3.95–3.99 (t, 2H, CH_2_, *J* = 6 Hz), 6.30–6.38 (m, 2H, ArH), 6.99–7.02 (d, 2H, ArH, *J* = 9 Hz), 7.64–7.67 (d, 2H, ArH, *J* = 9 Hz), 7.78–7.81 (d, 1H, ArH, *J* = 9 Hz), 8.37 (s, 1H, =CH), 10.20 (s, 1H, OH), 11.58 (s, 1H, NH), 12.44 (s, 1H, OH); ^13^C NMR (75 MHz, DMSO-*d_6_*): 10.82 (CH_3_), 22.44 (CH_2_), 69.57 (CH_2_), 103.31, 106.63, 107.80, 115.25, 127.06, 129.24, 129.98 (9C_ar_), 148.40 (=CH), 160.82, 162.80, 163.07 (3C_ar_), 165.81 (C=O).

*N*-[(4-butoxyphenyl)methylidene]-2,4-dihydroxybenzohydrazide (**7**)

CAS: 405220-47-5; light yellow powder; yield: 98%; M.p.: 246 °C; ^1^H NMR (300 MHz, DMSO-*d_6_*): 0.91–0.96 (t, 3H, CH_3_, *J* = 6 Hz, *J* = 9 Hz), 1.37–1.50 (m, 2H, CH_2_), 1.66–1.75 (m, 2H, CH_2_), 3.99–4.03 (t, 2H, CH_2_, *J* = 6 Hz), 6.30–6.38 (m, 2H, ArH), 6.99–7.02 (d, 2H, ArH, *J* = 9 Hz), 7.64–7.67 (d, 2H, ArH, *J* = 9 Hz), 7.78–7.81 (d, 1H, ArH, *J* = 9 Hz), 8.36 (s, 1H, =CH), 10.21 (s, 1H, OH), 11.58 (s, 1H, NH), 12.45 (s, 1H, OH); ^13^C NMR (75 MHz, DMSO-*d_6_*): 14.15 (CH_3_), 19.17 (CH_2_), 31.14 (CH_2_), 67.79 (CH_2_), 103.31, 106.62, 107.80, 115.25, 127.05, 129.23, 129.98 (9C_ar_), 148.41 (=CH), 160.83, 162.80, 163.07 (3C_ar_), 165.80 (C=O).

*N*-[(2-chloro-3-methoxyphenyl)methylidene]-2,4-dihydroxybenzohydrazide (**8**)

Yellow powder; yield: 58%; M.p.: 252 °C; ^1^H NMR (300 MHz, DMSO-*d_6_*): 3.88 (s, 3H, OCH_3_), 6.31–6.40 (m, 2H, ArH), 7.19–7.21 (m, 1H, ArH), 7.35–7.40 (t, 1H, ArH, *J* = 6 Hz, *J* = 9 Hz), 7.58–7.61 (d, 1H, ArH, *J* = 9 Hz), 7.80–7.83 (d, 1H, ArH, *J* = 9 Hz), 8.86 (s, 1H, =CH), 10.27 (s, 1H, OH), 11.92 (s, 1H, NH), 12.34 (s, 1H, OH); ^13^C NMR (75 MHz, DMSO-*d_6_*): 56.77 (OCH_3_), 103.34, 106.36, 107.92, 114.11, 118.77, 122.17, 128.31, 130.05, 133.13 (9C_ar_), 144.61 (=CH), 155.41, 163.13, 163.39 (3C_ar_), 166.32 (C=O).

*N*-[(3-chloro-4-methoxyphenyl)methylidene]-2,4-dihydroxybenzohydrazide (**9**)

CAS: 2488421-87-8; light yellow powder; yield: 63%; M.p.: 256 °C; ^1^H NMR (600 MHz, DMSO-*d_6_*): 3.92 (s, 3H, OCH_3_), 6.32–6.33 (d, 1H, ArH, *J* = 6 Hz), 6.37–6.38 (m, 1H, ArH), 7.24–7.25 (d, 1H, ArH, *J* = 6 Hz), 7.67–7.69 (m, 1H, ArH), 7.79–7.81 (m, 2H, ArH), 8.36 (s, 1H, =CH), 10.24 (s, 1H, OH), 11.70 (s, 1H, NH), 12.36 (s, 1H, OH); ^13^C NMR (150 MHz, DMSO-*d_6_*): 56.81 (OCH_3_), 103.31, 106.68, 107.90, 113.46, 122.13, 128.11, 128.28, 128.38, 130.19 (9C_ar_), 146.83 (=CH), 156.33, 162.66, 163.16 (3C_ar_), 165.83 (C=O).

*N*-[(3-bromo-4-methoxyphenyl)methylidene]-2,4-dihydroxybenzohydrazide (**10**)

CAS: 2489951-64-4; light yellow powder; yield: 81%; M.p.: 260 °C; ^1^H NMR (300 MHz, DMSO-*d_6_*): 3.90 (s, 3H, OCH_3_), 6.32–6.39 (m, 2H, ArH), 7.20–7.23 (d, 1H, ArH, *J* = 9 Hz), 7.67–7.68 (m, 1H, ArH), 7.79–7.82 (m, 2H, ArH), 8.35 (s, 1H, =CH), 10.22 (s, 1H, OH), 11.68 (s, 1H, NH), 12.34 (s, 1H, OH); ^13^C NMR (75 MHz, DMSO-*d_6_*): 56.75 (OCH_3_), 103.32, 106.69, 107.90, 113.40, 122.14, 128.10, 128.26, 128.36, 133.19 (9C_ar_), 146.83 (=CH), 156.31, 162.66, 163.16 (3C_ar_), 165.84 (C=O).

*N*-[(3-bromo-4-hydroxyphenyl)methylidene]-2,4-dihydroxybenzohydrazide (**11**)

CAS: 2488365-63-3; cream powder; yield: 98%; M.p.: 232 °C; ^1^H NMR (600 MHz, DMSO-*d_6_*): 6.22–6.27 (m, 1H, ArH), 6.31–6.32 (d, 1H, ArH, *J* = 6 Hz), 6.36–6.37 (m, 1H, ArH), 7.02–7.04 (d, 1H, ArH, *J* = 12 Hz), 7.56–7.58 (m, 1H, ArH), 7.78–7.80 (d, 1H, ArH, *J* = 6 Hz), 8.31 (s, 1H, =CH), 10.23 (s, 1H, OH), 10.82 (s, 1H, OH), 11.63 (s, 1H, NH), 12.40 (s, 1H, OH); ^13^C NMR (150 MHz, DMSO-*d_6_*): 103.31, 106.64, 107.51, 107.86, 110.29, 117.03, 127.40, 128.42, 130.09, 131.93 (10C_ar_), 147.15 (=CH), 156.36, 165.80 (2C_ar_), 169.12 (C=O).

*N*-[(3-ethoxy-4-hydroxyphenyl)methylidene]-2,4-dihydroxybenzohydrazide (**12**)

CAS: 405151-65-7; cream powder; yield: 96%; M.p.: 240 °C; ^1^H NMR (600 MHz, DMSO-*d_6_*): 1.36–1.38 (t, 3H, CH_3_, *J* = 6 Hz), 4.06–4.09 (q, 2H, CH_2_, *J* = 6 Hz, *J* = 6 Hz), 6.30–6.31 (d, 1H, ArH, *J* = 6 Hz), 6.35–6.37 (m, 1H, ArH), 6.85–6.87 (d, 1H, ArH, *J* = 12 Hz), 7.09–7.11 (m, 1H, ArH), 7.30–7.31 (m, 1H, ArH), 7.78–7.79 (d, 1H, ArH, *J* = 6 Hz), 8.31 (s, 1H, =CH), 9.51 (s, 1H, OH), 10.20 (s, 1H, OH), 11.55 (s, 1H, NH), 12.47 (s, 1H, OH); ^13^C NMR (150 MHz, DMSO-*d_6_*): 15.20 (CH_3_), 64.37 (OCH_2_), 103.34, 106.71, 107.76, 110.93, 116.04, 118.79, 122.61, 126.06, 129.95, 136.40 (10C_ar_), 147.66 (=CH), 149.81, 162.86 (2C_ar_), 165.76 (C=O).

2,4-dihydroxy-*N*-[(4-propylphenyl)methylidene]benzohydrazide (**13**)

CAS: 2486306-75-4; cream powder; yield: 23%; M.p.: 197 °C; ^1^H NMR (600 MHz, DMSO-*d_6_*): 0.89–0.92 (t, 3H, CH_3_, *J* = 6 Hz, *J* = 12 Hz), 1.58–1.64 (m, 2H, CH_2_), 2.58–2.61 (t, 3H, CH_3_, *J* = 12 Hz, *J* = 6 Hz), 6.32–6.33 (d, 1H, ArH, *J* = 6 Hz), 6.36–6.38 (m, 1H, ArH), 7.28–7.30 (d, 2H, ArH, *J* = 12 Hz), 7.64–7.65 (d, 2H, ArH, *J* = 6 Hz), 7.80–7.81 (d, 1H, ArH, *J* = 6 Hz), 8.41 (s, 1H, =CH), 10.24 (s, 1H, OH), 11.65 (s, 1H, NH), 12.40 (s, 1H, OH); ^13^C NMR (150 MHz, DMSO-*d_6_*): 14.09 (CH_3_), 24.36 (CH_2_), 37.59 (CH_2_), 103.32, 106.64, 107.87, 127.61, 129.33, 130.09, 132.26 (9C_ar_), 145.09 (=CH), 148.55, 162.78, 163.15 (3C_ar_), 165.89 (C=O).

*N*-[(3-ethoxy-4-methoxyphenyl)methylidene]-2,4-dihydroxybenzohydrazide (**14**)

CAS: 2488365-65-5; light yellow powder; yield: 70%; M.p.: 230 °C; ^1^H NMR (600 MHz, DMSO-*d_6_*): 1.35–1.38 (t, 3H, CH_3_, *J* = 12 Hz, *J* = 6 Hz), 3.82 (s, 3H, OCH_3_), 4.05–4.08 (q, 2H, CH_2_, *J* = 6 Hz, *J* = 6 Hz), 6.30–6.31 (d, 1H, ArH, *J* = 6 Hz), 6.35–6.37 (m, 1H, ArH), 7.03–7.05 (d, 1H, ArH, *J* = 12 Hz), 7.21–7.22 (m, 1H, ArH), 7.33–7.34 (m, 1H, ArH), 7.78–7.80 (d, 1H, ArH, *J* = 12 Hz), 8.35 (s, 1H, =CH), 10.22 (s, 1H, OH), 11.61 (s, 1H, NH), 12.42 (s, 1H, OH); ^13^C NMR (150 MHz, DMSO-*d_6_*): 15.20 (CH_3_), 56.01 (OCH_3_), 64.19 (OCH_2_), 103.33, 106.71, 107.80, 109.77, 112.05, 122.36, 126.86, 127.33, 130.06 (9C_ar_), 148.74 (=CH), 151.39, 158.83, 163.07 (3C_ar_), 165.75 (C=O).

*N*-[(2-bromo-3-hydroxy-4-methoxyphenyl)methylidene]-2,4-dihydroxybenzohydrazide (**15**)

CAS: 2488437-06-3; light yellow powder; yield: 39%; M.p.: 244 °C; ^1^H NMR (600 MHz, DMSO-*d_6_*): 3.89 (OCH_3_), 6.30–6.31 (d, 1H, ArH, *J* = 6 Hz), 6.36–6.38 (m, 1H, ArH), 7.10–7.11 (d, 1H, ArH, *J* = 6 Hz), 7.50–7.51 (d, 1H, ArH, *J* = 6 Hz), 7.81–7.82 (d, 1H, ArH, *J* = 6 Hz), 8.75 (s, 1H, =CH), 9.68 (s, 1H, OH), 10.26 (s, 1H, OH), 11.83 (s, 1H, NH), 12.49 (s, 1H, OH); ^13^C NMR (150 MHz, DMSO-*d_6_*): 56.74 (OCH_3_), 103.34, 106.25, 107.80, 111.50, 118.22, 121.55, 126.27, 129.85 (8C_ar_), 144.23 (=CH), 147.64. 150.14, 158.44, 163.28 (4C_ar_), 166.29 (C=O).

2,4-dihydroxy-*N*-[(4-hydroxy-3-iodo-5-methoxyphenyl)methylidene]benzohydrazide (**16**)

CAS: 2489894-46-2; light yellow powder; yield: 28%; M.p.: 255 °C; ^1^H NMR (600 MHz, DMSO-*d_6_*): 3.88 (s, 3H, OCH_3_), 6.31–6.32 (d, 1H, ArH, *J* = 12 Hz), 6.36–6.38 (m, 1H, ArH), 7.33–7.34 (m, 1H, ArH), 7.61–7.62 (m, 1H, ArH), 7.79–7.80 (d, 1H, ArH, *J* = 6 Hz), 8.28 (s, 1H, =CH), 10.08 (s, 1H, OH), 10.22 (s, 1H, OH), 11.64 (s, 1H, NH), 12.37 (s, 1H, OH); ^13^C NMR (150 MHz, DMSO-*d_6_*): 56.59 (OCH_3_), 84.90, 103.32, 106.73, 107.86, 109.54, 128.00, 130.14, 130.71 (8C_ar_), 147.29 (=CH), 147.78, 148.85, 162.64, 163.11 (4C_ar_), 165.74 (C=O).

*N*-[(3,5-dibromo-4-hydroxyphenyl)methylidene]-2,4-dihydroxybenzohydrazide (**17**)

CAS: 405226-82-6; light yellow powder; yield: 66%; M.p.: 260 °C; ^1^H NMR (600 MHz, DMSO-*d_6_*): 6.24–6.28 (m, 1H, ArH), 6.32–6.33 (d, 1H, ArH, *J* = 6 Hz), 6.36–6.38 (m, 1H, ArH), 7.63–7.64 (d, 1H, ArH, *J* = 6 Hz), 7.79–7.80 (d, 1H, ArH, *J* = 6 Hz), 7.91 (s, 1H, =CH), 8.29 (s, 1H, OH), 10.25 (s, 1H, OH), 11.76 (s, 1H, NH), 12.30 (s, 1H, OH); ^13^C NMR (150 MHz, DMSO-*d_6_*): 103.31, 107.94, 112.69, 129.32, 130.28, 131.14, 143.90 (9C_ar_), 145.35 (=CH), 152.61, 162.60, 163.22 (3C_ar_), 165.83 (C=O).

2,4-dihydroxy-*N*-[(2-hydroxy-3,5-diiodophenyl)methylidene]benzohydrazide (**18**)

CAS: 478379-97-4; yellow powder; yield: 57%; M.p.: 220 °C; ^1^H NMR (600 MHz, DMSO-*d_6_*): 6.35–6.36 (d, 1H, ArH, *J* = 6 Hz), 6.40–6.42 (m, 1H, ArH), 7.79–7.80 (d, 1H, ArH, *J* = 6 Hz), 7.87–7.88 (m, 1H, ArH), 8.06–8.07 (d, 1H, ArH), 8.46 (s, 1H, =CH), 10.35 (s, 1H, OH), 12.00 (s, 1H, NH), 12.22 (s, 1H, OH), 12.92 (s, 1H, OH); ^13^C NMR (150 MHz, DMSO-*d_6_*): 82.52, 88.23, 103.31, 108.30, 120.87, 128.86, 130.68, 139.23 (8C_ar_), 146.96 (=CH), 147.54, 157.07, 162.35, 163.62 (4C_ar_), 165.44 (C=O).

2,4-dihydroxy-*N*-[(2-nitrophenyl)methylidene]benzohydrazide (**19**)

CAS: 304481-61-06; orange powder; yield: 68%; M.p. 238–240 °C; ^1^H NMR (600 MHz, DMSO-*d_6_*): 6.32–6.33 (d, 1H, ArH, *J* = 6 Hz), 6.37–6.39 (m, 1H, ArH), 7.68–7.71 (m, 1H, ArH), 7.82–7.85 (m, 2H, ArH), 8.09–8.13 (m, 2H, ArH), 8.85 (s, 1H, =CH), 10.31 (s, 1H, OH), 12.05 (s, 1H, NH, 12.29 (s, 1H, OH); ^13^C NMR (150 MHz, DMSO-*d_6_*): 103.34, 106.43, 107.94, 125.15, 128.45, 129.10, 130.25, 131.18, 134.21, 142.52 (10C_ar_), 148.72 (=CH), 163.03, 163.43 (2C_ar_), 166.32 (C=O).

2,4-dihydroxy-*N*-[(3-nitrophenyl)methylidene]benzohydrazide (**20**)

CAS: 304481-71-8; light yellow powder; yield: 72%; M.p. 246–248 °C; ^1^H NMR (600 MHz, DMSO-*d_6_*): 6.34–6.35 (d, 1H, ArH, *J* = 6 Hz), 6.36–6.40 (m, 1H, ArH), 7.75–7.78 (m, 2H, ArH), 7.81–7.83 (d, 1H, ArH, *J* = 12 Hz), 8.15–8.18 (d, 1H, ArH, *J* = 18 Hz), 8.26–8.28 (m, 1H, ArH), 8.56 (s, 1H, =CH), 10.29 (s, 1H, OH), 11.91 (s, 1H, NH), 12.20 (s, 1H, OH); ^13^C NMR (150 MHz, DMSO-*d_6_*): 103.31, 106.84, 108.04, 121.42, 124.72, 130.55, 130.94, 133.82, 136.60, 145.82 (10C_ar_), 148.70 (=CH), 162.43, 163.31 (2C_ar_), 165.85 (C=O).

2,4-dihydroxy-*N*-[(4-nitrophenyl)methylidene]benzohydrazide (**21**)

CAS: 304481-38-7; yellow powder; yield: 59%; M.p. 252–254 °C; ^1^H NMR (600 MHz, DMSO-*d_6_*): 6.34–6.35 (d, 1H, ArH, *J* = 6 Hz), 6.38–6.40 (m, 1H, ArH), 7.81–7.82 (d, 1H, ArH, *J* = 6 Hz), 7.99–8.00 (d, 2H, ArH, *J* = 6 Hz), 8.31–8.32 (d, 2H, ArH, *J* = 6 Hz), 8.54 (s, 1H, =CH), 10.30 (s, 1H, OH), 11.98 (s, 1H, NH), 12.16 (s, 1H, OH); ^13^C NMR (150 MHz, DMSO-*d_6_*): 103.31, 106.90, 108.07, 124.56, 128.47, 130.62, 141.06, 145.72 (10C_ar_), 148.31 (=CH), 162.44, 163.37 (2C_ar_), 165.85 (C=O).

*N*-[(2-chlorophenyl)methylidene]-2,4-dihydroxybenzohydrazide (**22**)

CAS: 304481-37-6; light yellow powder; yield: 63%; M.p. 244–246 °C; ^1^H NMR (600 MHz, DMSO-*d_6_*): 6.32–6.33 (d, 1H, ArH, *J* = 6 Hz), 6.38–6.39 (m, 1H, ArH), 7.43–7.47 (m, 2H, ArH), 7.53–7.54 (m, 1H, ArH), 7.81–7.83 (d, 1H, ArH, *J* = 12 Hz), 8.02–8.03 (d, 1H, ArH, *J* = 6 Hz), 8.85 (s, 1H, =CH), 10.30 (s, 1H, OH), 11.94 (s, 1H, NH), 12.36 (s, 1H, OH); ^13^C NMR (150 MHz, DMSO-*d_6_*): 103.35, 106.34, 107.92, 127.38, 128.09, 130.01, 130.40, 132.00, 133.70, 141.26 (10C_ar_), 144.29 (=CH), 163.13, 163.39 (2C_ar_), 166.32 (C=O).

*N*-[(2-bromophenyl)methylidene]-2,4-dihydroxybenzohydrazide (**23**)

CAS: 405220-19-1; white powder; yield: 75%; M.p. 243–245 °C; ^1^H NMR (600 MHz, DMSO-*d_6_*): 6.32–6.33 (d, 1H, ArH, *J* = 6 Hz), 6.38–6.39 (m, 1H, ArH), 7.36–7.39 (m, 1H, ArH), 7.46–7.48 (m, 1H, ArH), 7.69–7.71 (m, 1H, ArH), 7.82–7.83 (d, 1H, ArH, *J* = 6 Hz), 7.99–8.01 (d, 1H, ArH, *J* = 12 Hz), 8.80 (s, 1H, =CH), 10.31 (s, 1H, OH), 11.98 (s, 1H, NH), 12.38 (s, 1H, OH); ^13^C NMR (150 MHz, DMSO-*d_6_*): 103.35, 106.29, 107.91, 124.07, 127.76, 128.57, 129.99, 132.25, 133.45, 133.64 (10C_ar_), 146.63 (=CH), 163.19, 163.40 (2C_ar_), 166.40 (C=O).

*N*-[(2-fluorophenyl)methylidene]-2,4-dihydroxybenzohydrazide (**24**)

CAS: 405220-67-9; white powder; yield: 78%; M.p. 251–253 °C; ^1^H NMR (600 MHz, DMSO-*d_6_*): 6.32–6.33 (d, 1H, ArH, *J* = 6 Hz), 6.37–6.39 (m, 1H, ArH), 7.29–7.33 (m, 2H, ArH), 7.49–7.52 (m, 1H, ArH), 7.80–7.81 (d, 1H, ArH, *J* = 6 Hz), 7.94–7.96 (m, 1H, ArH), 8.68 (s, 1H, =CH), 10.29 (s, 1H, OH), 11.84 (s, 1H, NH), 12.33 (s, 1H, OH); ^13^C NMR (150 MHz, DMSO-*d_6_*): 103.33, 106.45, 107.94, 116.42, 116.56, 122.21, 125.42, 126.83, 130.07, 132.55 (10C_ar_), 141.12 (=CH), 162.10, 162.96 (2C_ar_), 166.12 (C=O).

### 4.3. Microbiology—Antimicrobial Activity

The examined compounds **1**–**24** were screened in vitro for antibacterial and antifungal activities using the broth microdilution method, according to the procedure reported earlier by our research group [24,25,31,32] and European Committee on Antimicrobial Susceptibility Testing (EUCAST) [50] and Clinical and Laboratory Standards Institute (CLSI) guidelines [51] against a panel of reference and clinical or saprophytic strains of microorganisms. Detailed procedures are presented in the Supplementary Materials.

### 4.4. Cell Cultures

The in vitro studies were performed on the reference line HEK-293 (human embryonic kidney cells, ATCC^®^ CRL-1573™) and three human cancer cell lines: 769-P (adenocarcinoma, renal cells, ATCC^®^ CRL-1933™) and HepG2 (carcinoma, hepatocellular, ATCC^®^ HB-8065™), H1563 (adenocarcinoma, non-smallcell lung cancer, ATCC^®^ CRL-5875™), LN-229 (glioblastoma, ATCC^®^ CRL-2611™). Cells from the 769-P and H1563 cell lines were cultured in RPMI-1640 growth medium (PAN Biotech GmbH, Aidenbach, Bayern, Germany), cells from the HEK-293 and HepG2 cell lines were cultured in EMEM growth medium (PAN Biotech GmbH, Aidenbach, Bayern, Germany), and cells from the LN-229 cell line were cultured in DMEM growth medium (PAN Biotech GmbH, Aidenbach, Bayern, Germany) in sterile 75 cm^2^ culture phalcones. Fetal bovine serum (PAN Biotech GmbH, Aidenbach, Bayern, Germany) at 10% of growth medium and antibiotics (Sigma-Aldrich, Poland) (2.5 µg/mL amphotericin B, 100 µg/mL penicillin, and 100 µg/mL streptomycin) were added to all media. Cells were cultured at 37 °C in an incubator with 5% CO_2_ flow until a cell layer was formed. Following this, the medium was removed, and the cells were washed with 0.25% trypsin solution with 2.21 mM EDTA (Corning Incorporated, Corning, NY, USA) in order to completely remove fetal bovine serum containing trypsin inhibitor. Finally, 6 mL of complete growth medium was added, and the cells were aspirated by gentle pipetting.

### 4.5. Breeding and Egg Collection of Danio rerio

Wild-type AB strain zebrafish (*Danio rerio*) were housed in the Experimental Medicine Centre of Medical University of Lublin (Lublin, Poland), where all the experimental procedures on embryos and larvae were carried out. The animals were kept on a recirculating water supply at 26 ± 1 °C and a 10/14 dark–light cycle, and they were fed with artemia and commercial feed [39]. *Danio rerio* embryos were obtained from females between 4 and 15 months of age. No signs of disease or infection were observed in the fish which were used for spawning, and they were not treated with pharmaceuticals in the 2 months prior. In order to avoid genetic bias in the study, eggs were randomly collected from at least three breeding groups, rinsed, and placed in Petri dishes in the cultured medium, which for *Danio rerio* is E3 solution. It does not affect the development of fish and contains 0.17 mM KCl, 0.33 mM CaCl_2_, 0.33 mM MgSO_4_, and 5 mM NaCl, with the addition of a fresh solution of methylene blue as an antimicrobial and antifungal agent. In accordance with the legislation of the European Union and Poland, the experiments performed on the earliest life-stages of *Danio rerio* until 120 h post-fertilization (120 hpf), not defined as protected, are not subject to regulations for animal experimentation.

### 4.6. Cytotoxicity Assay

The determination of inhibitory concentration (IC_50_) in the HEK-293, 769-P, HepG2, H1563, and LN-229 cell cultures for selected hydrazide–hydrazones was performed using the MTT assay procedure. This test was performed according to the MTT Assay ECVAM n°17 protocol manual prepared by the European Centre for the Validation of Alternative Methods (ECVAM), an entity that aims to reduce in vivo testing in favor of in vitro and in silico methods [23]. To 96-well sterile plates, 100 μL of cell suspensions, prepared in the appropriate medium at a concentration of 3 × 10^5^ cells/mL, was added using a multichannel pipette. To achieve cell adhesion to the bottom of the plates, cells were incubated for 24 h at 37 °C with 5% CO_2_ flow and 90% humidity. To determine the growth-inhibitory concentration, the solutions of the tested substances (**19**–**24**) were prepared at concentrations ranging from 1 µM to 500 µM, added individually to the wells following the rule of 8 wells per concentration, and the plates were again incubated for 24 h at 37 °C and with 5% CO_2_ addition. We also prepared a background control (200 μL of the respective growth medium) and negative control (100 μL of cells in the appropriate growth medium + 100 μL of the growth medium), which provided information on the amount of formazan formed in the untreated cells. After a 24 h incubation period, the solutions were extracted from the cells; 190 μL of growth medium and 10 μL of 5 mg/mL MTT solution (Invitrogen, Waltham, MA, USA) were added each and incubated for 4 h. After this time, all medium was removed, 100 μL of DMSO (Corning Incorporated, Corning, NY, USA) was added to each well, and absorbance measurements were performed at 550 nm using a Power Weave xs spectrophotometric microplate reader (BioTek, Winooski, VT, USA). The decrease in absorbance is equivalent to the decrease in MTT formazan production, which is due to the decrease in mitochondrial dehydrogenase activity and indicates a cytotoxic effect from the tested substances. Cell viability is expressed as a percentage as the ratio of the absorbance of test substance-treated cells to the untreated cells. The experiments were repeated three times.

### 4.7. Acute Toxicity Study in the Zebrafish Embryo Model

The acute toxicity assessment of the synthesized hydrazide–hydrazones was performed using Guideline 236 recommended by the Organization for Economic Cooperation and Development (OECD) [53]. They address the testing of chemicals using the Fish Embryo Acute Toxicity (FET) test with the species *Danio rerio*.

Immediately after fertilization, eggs were placed in sterile 24-well plates and exposed for 96 h to at least five concentrations of the test chemical, ranging from 1 to 500 µM and prepared ex tempore on each day of exposure, according to the principle of one egg per well and one concentration per plate. In each plate, 20 wells were filled with the solutions of the tested substance, and 4 wells of the last column were a negative internal control with E3 medium. Each well was filled with 2 mL of solution. Positive control plates were also prepared according to this scheme with 4 mg/L of 3,4-dichloroaniline (Sigma-Aldrich, Poznań, Poland) and a negative control with E3, and a negative solvent control with 1% DMSO, which was used as a solvent for the tested substances. At four time points, microscopic observations were made every 24 h. As indicators of mortality (so-called terminal observations; when at least one of these occurs, embryonic death is declared), the following were recorded: the coagulation of embryos, a lack of somite formation, a lack of detachment of the tail from the yolk sac and a lack of a heartbeat, which was observed from the 48th hour of the study. To confirm the absence of a heartbeat, a microscopic observation was conducted at 80× magnification for one minute. Then, the solutions of the tested chemicals, to maintain their constant composition throughout the test, were replaced with fresh ones, and the plates were incubated for another 24 h in an incubator at 26 ± 1 °C. At the end of the 96 h exposure, acute toxicity was determined based on the observations by calculating the half-maximal lethal concentration value (LC_50_), which means the concentration of molecules that causes the death of 50% of zebrafish embryos/larvae during the experimental period (96 h). In addition, a quantitative and qualitative summary of the morphological and physiological abnormalities that occurred in the tested *Danio rerio* embryos and larvae was also made. These included yolk sac edema, pericardial edema, spinal flexure, head deformities, tail deformities, developmental delay defined as a lack of hatching success for 96 hpf larvae, and also bradycardia. All the microscopic observations were performed using a Stemi 508 stereomicroscope (Carl Zeiss Microscopy GmbH, Jena, Germany). The evaluation of the heart rate of the *Danio rerio* larvae in the control and test trials after 96 h of exposure to different concentrations of the tested substances, E3 medium, and solvent (1% DMSO) was performed by counting the number of heartbeats per minute, using a Stemi 508 stereomicroscope.

### 4.8. Statistical Analysis

The IC_50_ and LC_50_ values for the newly obtained hydrazide–hydrazones were derived from the respective concentration–response curves, where the percentage cytotoxicity or mortality after 24 h of incubation or 96 h of exposure, respectively, is plotted versus concentration on a logarithmic scale. Regression analysis was performed with Excel, and the above-mentioned values were obtained from the linear type of regression analysis. The statistical analysis (distribution of data) was performed using GraphPad Prism software (version 5.01 for Windows; GraphPad Software Inc., San Diego, CA, USA). The results obtained from the heart rate study of 96 hpf zebrafish larvae were subjected to statistical analysis using one-way analysis of variance (ANOVA) and a post hoc Bonferroni test. The significance level was taken as *p* < 0.05.

## 5. Conclusions

Our obtained results of bioactivity assays confirmed that among the synthesized hydrazide–hydrazones of 2,4-dihydroxybenzoic acid (**1**–**24**), we found both promising antibacterial agents (**2**, **9**, **10**, **11**, **17**, and especially **18**) and substances with very significant antiproliferative activity and low toxicity (**19**–**24**).

## Figures and Tables

**Table 1:** Yield of the reactions, reaction time, and melting points of hydrazide–hydrazones of 2,4-dihydroxybenzoic acid.

Compound Number	Yield (%)	Reaction Time (min)	Melting Point (°C)	Compound Number	Yield (%)	Reaction Time (min)	Melting Point (°C)
**1**	83	16	250	**13**	23	40	197
**2**	96	15	205	**14**	70	31	230
**3**	98	16	248	**15**	39	33	244
**4**	95	18	263	**16**	28	39	255
**5**	94	17	248	**17**	66	37	260
**6**	95	20	251	**18**	57	40	220
**7**	98	23	246	**19**	68	33	238–240
**8**	58	26	252	**20**	72	36	246–248
**9**	63	30	256	**21**	59	36	252–254
**10**	81	31	260	**22**	63	37	244–246
**11**	98	29	232	**23**	75	40	243–245
**12**	96	36	240	**24**	78	40	251–253

**Table 2:** The activity data of studied compounds **1**–**9** expressed as MIC (MBC or MFC) [µg/mL] and {MBC/MIC or MFC/MIC} values against the reference strains of bacteria and fungi.

Species		MIC (MBC or MFC) [µg/mL] and {MBC/MIC or MFC/MIC} Values of the Tested Compounds and Reference Medicines										
		1	2	3	5	6	7	9	CIP/VA * /NY **	NIT	CFX	APC
**Gram-positive bacteria**	*Staphylococcus* *aureus* ATCC 43300	1000 (>1000) {>1}	**250** **(500)** **{2}**	-	-	1000 (>1000) {>1}	-	**15.62** **(62.5)** **{4}**	0.24 (0.24) {1}	7.81 (15.62)	nd	nd
	*Staphylococcus* *aureus* ATCC 29213	1000 (>1000) {>1}	**250** **(250)** **{1}**	-	1000 (>1000) {>1}	1000 (>1000) {>1}	1000 (>1000) {>1}	**31.25** **(31.25)** **{1}**	0.48 (0.48) {1}	nd	nd	nd
	*Staphylococcus* *epidermidis* ATCC 12228	-	250 (500) {**2**}	-	-	1000 (>1000) {>1}	**500** **(1000)** **{2}**	**62.5** **(125)** **{2}**	0.12 (0.12) {1}	3.91 (7.81)	0.24	nd
	*Enterococcus* *faecalis* ATCC 29212	1000 (>1000) {>1}	**500** **(500)** **{1}**	-	1000 (>1000) {>1}	-	-	**125** **(500)** **{4}**	0.98 * (1.95) {2}	nd	nd	nd
	*Micrococcus* *luteus* ATCC 10240	500 (>1000) {>2}	31.25 (250) {8}	500 (>1000) {>2}	500 (>1000) {>2}	1000 (>1000) {>1}	1000 (>1000) {>1}	**125** **(500)** **{4}**	0.98 (1.95) {2}	62.5 (62.5)	0.98	nd
	*Bacillus* *subtilis* ATCC 6633	500 (>1000) {>2}	62.5 (500) {8}	-	500 (>1000) {>2}	1000 (>1000) {>1}	-	**125** **(500)** **{4}**	0.03 (0.03) {1}	3.91 (3.91)	15.62	62.5
	*Bacillus* *cereus* ATCC 10876	-	125 (250) {**2**}	-	500 (>1000) {>2}	1000 (>1000) {>1}	1000 (>1000) {>1}	**15.62** **(62.5)** **{4}**	0.06 (0.12) {2}	7.81 (15.62)	31.25	nd
**Gram-negative bacteria**	*Bordetella* *bronchiseptica* ATCC 4617	-	1000 (>1000) {>1}	-	-	-	-	-	0.98 (0.98) {1}	125 (>1000)	nd	nd
	*Klebsiella* *pneumoniae* ATCC 13883	-	1000 (>1000) {>1}	-	-	-	-	-	0.12 (0.24) {2}	15.62 (31.25)	nd	nd
	*Proteus* *mirabilis* ATCC 12453	-	1000 (>1000) {>1}	-	-	-	-	-	0.03 (0.03) {1)	62.5 (125)	nd	nd
	*Salmonella* *typhimurium* ATCC 14028	-	1000 (>1000) {>1}	-	-	-	-	-	0.06 (0.06) {1}	31.25 (62.5)	nd	nd
	*Escherichia* *coli* ATCC 25922	-	1000 (>1000) {>1}	-	-	-	-	-	0.004 (0.008) {2}	7.81 (15.62)	nd	nd
	*Pseudomonas* *aeruginosa* ATCC 9027	-	-	-	-	-	-	-	0.48 (0.98) {2}	nd	nd	nd
**Fungi**	*Candida* *albicans* ATCC 2091	-	-	-	500 (>1000) {>2}	500 (>1000) {>2}	500 (>1000) {>2}	-	0.24 ** (0.24) {1}	na	na	na
	*Candida* *albicans* ATCC 10231	-	-	-	1000 (>1000) {>1}	1000 (>1000) {>1}	1000 (>1000) {>1}	-	0.48 ** (0.48) {1}	na	na	na
	*Candida* *parapsilosis* ATCC 22019	-	500 (>1000) {>2}	-	-	1000 (>1000) {>1}	-	-	0.24 ** (0.48) {2}	na	na	na
	*Candida* *glabrata* ATCC 90030	-	**250** **(500)** **{2}**	-	-	-	-	-	0.24 ** (0.48) {2}	na	na	na
	*Candida* *krusei* ATCC 14243	-	-	-	-	-	-	-	0.24 ** (0.24) {1}	na	na	na

‘-’, no activity; na, not applicable; nd, not determined. The standard antimicrobial medicines used as reference substances: ciprofloxacin (CIP), nitrofurantoin (NIT), cefuroxime (CFX), and ampicillin (APC) for bacteria (except enterococci), vancomycin (VA *) for enterococci, and nystatin (NY **) for fungi; compounds with bactericidal (MBC/MIC ≤ 4) or fungicidal (MFC/MIC ≤ 4) effects are marked with bold font.

**Table 3:** The activity data of studied compounds **10**–**18** expressed as MIC (MBC or MFC) [µg/mL] and {MBC/MIC or MFC/MIC} values against the reference strains of bacteria and fungi.

Species		MIC (MBC or MFC) [µg/mL] and {MBC/MIC or MFC/MIC} Values of the Tested Compounds and Reference Medicines											
		10	11	13	14	15	16	17	18	CIP/VA * /NY **	NIT	CFX	APC
**Gram-positive bacteria**	*Staphylococcus* *aureus* ATCC 43300	31.25 (500) {16}	**500** **(1000)** **{2}**	1000 (>1000) {>1}	-	-	-	62.5 (>1000) {>16}	3.91 (31.25) {8}	0.24 (0.24) {1}	7.81 (15.62)	nd	nd
	*Staphylococcus* *aureus* ATCC 29213	15.62 (250) {16}	250 (>1000) {>4}	1000 (>1000) {>1}	-	-	1000 (>1000) {>1}	31.25 (>1000) {32}	**7.81** **(15.62)** **{2}**	0.48 (0.48) {1}	nd	nd	nd
	*Staphylococcus* *epidermidis* ATCC 12228	1000 (1000) {>1}	**500** **(1000)** **{2}**	1000 (>1000) {>1}	-	1000 (>1000) {>1}	1000 (>1000) {>1}	250 (>1000) {>8}	0.98 (7.81) {8}	0.12 (0.12) {1}	3.91 (7.81)	0.24	nd
	*Enterococcus* *faecalis* ATCC 29212	**250** **(1000)** **{4}**	500 (>1000) {>2}	1000 (>1000) {>1}	-	-	1000 (>1000) {>1}	500 (>1000) {>1}	**3.91** **(15.62)** **{4}**	0.98 * (1.95) {2}	nd	nd	nd
	*Micrococcus* *luteus* ATCC 10240	250 (>1000) {>4}	125 (>1000) {>8}	1000 (>1000) {>1}	125 (>1000) {>8}	500 (>1000) {>2}	250 (>1000) {>4}	125 (>1000) {>8}	**0.48** **(1.95)** **{4}**	0.98 (1.95) {2}	62.5 (62.5)	0.98	nd
	*Bacillus* *subtilis* ATCC 6633	250 (>1000) {>4}	500 (>1000) {>2}	1000 (>1000) {>1}	-	-	250 (>1000) {>4}	125 (>1000) {>8}	**1.95** **(3.91)** **{2}**	0.03 (0.03) {1}	3.91 (3.91)	15.62	62.5
	*Bacillus* *cereus* ATCC 10876	**125** **(250)** **{2}**	250 (>1000) {>4}	1000 (>1000) {>1}	500 (>1000) {>2}	-	1000 (>1000) {>1}	250 (>1000) {>4}	**3.91** **(15.62)** **{4}**	0.06 (0.12) {2}	7.81 (15.62)	31.25	nd
**Gram-negative bacteria**	*Bordetella* *bronchiseptica* ATCC 4617	-	-	1000 (>1000) {>1}	**1000** **(1000)** **{1}**	-	-	1000 (>1000) {>1}	500 (>1000) {>2}	0.98 (0.98) {1}	125 (>1000)	nd	nd
	*Klebsiella* *pneumoniae* ATCC 13883	-	-	1000 (>1000) {>1}	-	-	-	1000 (>1000) {>1}	500 (>1000) {>2}	0.12 (0.24) {2}	15.62 (31.25)	nd	nd
	*Proteus* *mirabilis* ATCC 12453	-	-	1000 (>1000) {>1}	-	-	-	1000 (>1000) {>1}	500 (>1000) {>2}	0.03 (0.03) {1)	62.5 (125)	nd	nd
	*Salmonella* *typhimurium* ATCC 14028	-	-	1000 (>1000) {>1}	-	-	-	1000 (>1000) {>1}	1000 (>1000) {>1}	0.06 (0.06) {1}	31.25 (62.5)	nd	nd
	*Escherichia* *coli* ATCC 25922	-	-	1000 (>1000) {>1}	-	-	-	1000 (>1000) {>1}	500 (>1000) {>2}	0.004 (0.008) {2}	7.81 (15.62)	nd	nd
	*Pseudomonas* *aeruginosa* ATCC 9027	-	-	1000 (>1000) {>1}	-	-	-	-	1000 (>1000) {>1}	0.48 (0.98) {2}	nd	nd	nd
**Fungi**	*Candida* *albicans* ATCC 2091	-	-	-	-	-	250 (>1000) {>4}	1000 (>1000) {>1}	500 (>1000) {>2}	0.24 ** (0.24) {1}	na	na	na
	*Candida* *albicans* ATCC 10231	-	-	1000 (>1000) {>1}	500 (>1000) {>2}	-	1000 (>1000) {>1}	1000 (>1000) {>1}	1000 (>1000) {>1}	0.48 ** (0.48) {1}	na	na	na
	*Candida* *parapsilosis* ATCC 22019	-	-	1000 (>1000) {>1}	-	1000 (>1000) {>1}	-	-	500 (>1000) {>2}	0.24 ** (0.48) {2}	na	na	na
	*Candida* *glabrata* ATCC 90030	-	-	1000 (>1000) {>1}	-	-	-	-	1000 (>1000) {>1}	0.24 ** (0.48) {2}	na	na	na
	*Candida* *krusei* ATCC 14243	-	-	-	-	-	-	-	1000 (>1000) {>1}	0.24 ** (0.24) {1}	na	na	na

‘-’, no activity; na, not applicable; nd, not determined. The standard antimicrobial medicines used as reference substances: ciprofloxacin (CIP), nitrofurantoin (NIT), cefuroxime (CFX), and ampicillin (APC) for bacteria (except enterococci), vancomycin (VA *) for enterococci, and nystatin (NY **) for fungi; compounds with bactericidal (MBC/MIC ≤ 4) or fungicidal (MFC/MIC ≤ 4) effects are marked with bold font.

**Table 4:** The activity data of studied compounds **19**–**24** expressed as MIC (MBC or MFC) [µg/mL] and {MBC/MIC or MFC/MIC} values against the reference strains of bacteria and fungi.

Species		MIC (MBC or MFC) [µg/mL] and {MBC/MIC or MFC/MIC} Values of the Tested Compounds and Reference Medicines									
		19	20	21	22	23	24	CIP/VA * /NY **	NIT	CFX	APC
**Gram-positive bacteria**	*Staphylococcus* *aureus* ATCC 43300	500 (>1000) {>2}	-	500 (>1000) {>2}	-	-	-	0.24 (0.24) {1}	7.81 (15.62)	nd	nd
	*Staphylococcus* *aureus* ATCC 29213	1000 (>1000) {>1}	-	500 (>1000) {>2}	-	-	-	0.48 (0.48) {1}	nd	nd	nd
	*Staphylococcus* *epidermidis* ATCC 12228	500 (>1000) {>2}	-	500 (>1000) {>2}	1000 (>1000) {>1}	500 (>1000) {>2}	-	0.12 (0.12) {1}	3.91 (7.81)	0.24	nd
	*Enterococcus* *faecalis* ATCC 29212	500 (>1000) {>2}	-	500 (>1000) {>4}	-	-	1000 (>1000) {>1}	0.98 * (1.95) {2}	nd	nd	nd
	*Micrococcus* *luteus* ATCC 10240	1000 (>1000) {>1}	-	1000 (>1000) {>1}	-	-	1000 (>1000) {>1}	0.98 (1.95) {2}	62.5 (62.5)	0.98	nd
	*Bacillus* *subtilis* ATCC 6633	250 (>1000) {>4}	250 (>1000) {>4}	500 (>1000) {>2}	-	-	500 (>1000) {>2}	0.03 (0.03) {1}	3.91 (3.91)	15.62	62.5
	*Bacillus* *cereus* ATCC 10876	250 (>1000) {>4}	-	500 (>1000) {>2}	-	-	1000 (>1000) {>1}	0.06 (0.12) {2}	7.81 (15.62)	31.25	nd
**Gram-negative bacteria**	*Bordetella* *bronchiseptica* ATCC 4617	1000 (>1000) {>1}	-	-	-	-	-	0.98 (0.98) {1}	125 (>1000)	nd	nd
	*Klebsiella* *pneumonia* ATCC 13883	500 (>1000) {>1}	-	-	-	-	-	0.12 (0.24) {2}	15.62 (31.25)	nd	nd
	*Proteus* *mirabilis* ATCC 12453	1000 (>1000) {>1}	-	-	-	-	-	0.03 (0.03) {1)	62.5 (125)	nd	nd
	*Salmonella* *typhimurium* ATCC 14028	1000 (>1000) {>1}	-	-	-	-	-	0.06 (0.06) {1}	31.25 (62.5)	nd	nd
	*Escherichia* *coli* ATCC 25922	1000 (>1000) {>1}	-	-	-	-	-	0.004 (0.008) {2}	7.81 (15.62)	nd	nd
	*Pseudomonas* *aeruginosa* ATCC 9027	1000 (>1000) {>1}	-	-	-	-	-	0.48 (0.98) {2}	nd	nd	nd
**Fungi**	*Candida* *albicans* ATCC 2091	500 (>1000) {>2}	-	1000 (>1000) {>1}	1000 (>1000) {>1}	1000 (>1000) {>1}	1000 (>1000) {>1}	0.24 ** (0.24) {1}	na	na	na
	*Candida* *albicans* ATCC 10231	250 (>1000) {>4}	1000 (>1000) {>1}	500 (>1000) {>2}	1000 (>1000) {>1}	1000 (>1000) {>1}	1000 (>1000) {>1}	0.48 ** (0.48) {1}	na	na	na
	*Candida* *parapsilosis* ATCC 22019	250 (>1000) {>4}	1000 (>1000) {>1}	500 (>1000) {>2}	1000 (>1000) {>1}	1000 (>1000) {>1}	1000 (>1000) {>1}	0.24 ** (0.48) {2}	na	na	na
	*Candida* *glabrata* ATCC 90030	500 (>1000) {>2}	-	-	-	-	-	0.24 ** (0.48) {2}	na	na	na
	*Candida* *krusei* ATCC 14243	500 (>1000) {>2}	-	500 (>1000) {>2}	1000 (>1000) {>1}	-	1000 (>1000) {>1}	0.24 ** (0.24) {1}	na	na	na

‘-’, no activity; na, not applicable; nd, not determined. The standard antimicrobial medicines used as reference substances: ciprofloxacin (CIP), nitrofurantoin (NIT), cefuroxime (CFX), and ampicillin (APC) for bacteria (except enterococci), vancomycin (VA *) for enterococci, and nystatin (NY **) for fungi;

**Table 5:** The antiproliferative activity and selectivity of synthesized selected hydrazide–hydrazones in human cancer cell lines.

Compound	R	IC_50_ in HEK-293, µM	IC_50_ in 769-P, µM	Selectivity Index ^a^	IC_50_ in HepG2, µM	Selectivity Index ^a^	IC_50_ in H1563, µM	Selectivity Index ^a^	IC_50_ in LN-229, µM	Selectivity Index ^a^
**19**	2-NO_2_-Ph	1.49 × 10^5^	45.42	3.29 × 10^3^	>500.00	nd	65.57	2.27 × 10^3^	130.17	1.14 × 10^3^
**20**	3-NO_2_-Ph	1.34 × 10^4^	>500.00	nd	>500.00	nd	70.94	1.89 × 10^2^	>500.00	nd
**21**	4-NO_2_-Ph	4.45 × 10^10^	12.39	3.59 × 10^9^	7.81	5.69 × 10^9^	>500.00	nd	0.77	5.78 × 10^9^
**22**	2-Cl-Ph	314.68	262.48	1.20	nd	nd	216.73	1.45	110.53	2.85
**23**	2-Br-Ph	9.18 × 10^4^	>500.00	nd	nd	nd	101.14	9.08 × 10^2^	156.77	5.85 × 10^2^
**24**	2-F-Ph	386.73	>500.00	nd	>500.00	nd	199.69	1.94	199.32	1.94

R, substituent; nd, not determined. ^a^ Calculated as the ratio between the IC_50_ values in the non-tumor cell line HEK-293 and the respective cancer cell lines (769-P, HepG2, H1563, and LN-229).

**Table 6:** The acute toxicity of synthesized selected hydrazide–hydrazones assessed in the zebrafish embryo model.

Compound	R	LC_50_, µM	LC_50_, mg/L
**19**	2-NO_2_-Ph	1.88 × 10^5^	5.68 × 10^4^
**20**	3-NO_2_-Ph	3.48 × 10^4^	1.05 × 10^4^
**21**	4-NO_2_-Ph	370.00	110.00
**22**	2-Cl-Ph	56.56	16.44
**23**	2-Br-Ph	85.62	30.41
**24**	2-F-Ph	4.24 × 10^3^	1.16 × 10^3^

## Data Availability

Data are contained within the article or Supplementary Materials.

## Supplementary Materials

The supporting information can be downloaded at: https://www.mdpi.com/article/10.3390/ijms242417481/s1. References [66,67] are cited in the supplementary materials.
