# Supplementary material for: Design, Synthesis, and In Vitro and In Vivo Bioactivity Studies of Hydrazide–Hydrazones of 2,4-Dihydroxybenzoic Acid
Source: Int J Mol Sci. 2023 Dec 14;24(24):17481. doi: 10.3390/ijms242417481 (PMC10743905; doi:10.3390/ijms242417481)
Supplement: Supplementary file 1 [file ijms-24-17481-s001.zip › ijms-2680013-supplementary.pdf]

International Journal of *Molecular Sciences*

MDPI

Article

Design, Synthesis, and In Vitro and In Vivo Bioactivity Studies of Hydrazide–Hydrazones of 2,4-Dihydroxybenzoic Acid

Łukasz Popiołek [1,*], Monika Gawrońska-Grzywacz [2], Aleksandra Dziduch [1], Anna Biernasiuk [3], Iwona Piątkowska-Chmiel [2] and Mariola Herbet [2]

[1] Chair and Department of Organic Chemistry, Faculty of Pharmacy, Medical University of Lublin, 4A Chodźki Street, 20-093 Lublin, Poland; 54134@student.umlub.pl

[2] Chair and Department of Toxicology, Faculty of Pharmacy, Medical University of Lublin, 8B Jaczewskiego Street, 20-090 Lublin, Poland; monika.gawronska-grzywacz@umlub.pl (M.G.-G.); iwona.piatkowska-chmiel@umlub.pl (I.P.-C.); mariola.herbet@umlub.pl (M.H.)

[3] Chair and Department of Pharmaceutical Microbiology, Faculty of Pharmacy, Medical University of Lublin, 1 Chodźki Street, 20-093 Lublin, Poland; anna.biernasiuk@umlub.pl (A.B.)

* Correspondence: lukasz.popiolek@umlub.pl; Tel.: +48-81-448-72-43

SUPPLEMENTARY MATERIALS

In vitro antimicrobial activity assays - Methodology

The examined compounds **1** - **24** were screened *in vitro* for antibacterial and antifungal activities using the broth microdilution method according to procedure reported earlier by our research group [1-3] and European Committee on Antimicrobial Susceptibility Testing (EUCAST) [4] and Clinical and Laboratory Standards Institute (CLSI) guidelines [5] against a panel of reference and clinical or saprophytic strains of microorganisms, including Gram-positive bacteria, *Staphylococcus aureus* ATCC 43300 (Methicillin Resistant *S. aureus* – MRSA), *Staphylococcus aureus* ATCC 29213 (Methicillin Susceptible *S. aureus* – MSSA), *Staphylococcus epidermidis* ATCC 12228, *Enterococcus faecalis* ATCC 29212, *Micrococcus luteus* ATCC 10240, *Bacillus subtilis* ATCC 6633 and *Bacillus cereus* ATCC 10876), Gram-negative bacteria (*Bordetella bronchiseptica* ATCC 4617, *Escherichia coli* ATCC 25922, *Klebsiella pneumoniae* ATCC 13883, *Proteus mirabilis* ATCC 12453, *Salmonella typhimurium* ATCC 14028 and *Pseudomonas aeruginosa* ATCC 9027) and fungi belonging to yeasts (*Candida albicans* ATCC 10231, *Candida albicans* ATCC 2091, *Candida parapsilosis* ATCC 22019, *Candida glabrata* ATCC 90030 and *Candida krusei* ATCC 14243). The microorganisms belonging to ATCC came from American Type Culture Collection, routinely used for the evaluation of antimicrobials. All the used microbial cultures were first subcultured on nutrient agar or Sabouraud agar at 35°C for 18-24 h or 30°C for 24-48 h for bacteria and fungi, respectively.

The surface of Mueller-Hinton agar (for bacteria) and RPMI 1640 with MOPS (for fungi) were inoculated with the suspensions of bacterial or fungal species. Microbial suspensions were prepared in sterile saline with an optical density of McFarland standard scale 0.5. Samples containing examined compounds were dissolved in dimethyl sulfoxide (DMSO). Furthermore, bacterial and fungal suspensions were put onto Petri dishes with solid media containing 2 mg/mL of the tested compounds followed incubation at 37°C for 24h and 30°C for 48h for bacteria and fungi, respectively. The inhibition of microbial growth was judged by comparison with a control culture prepared without any sample tested. Ciprofloxacin, vancomycin, nitrofurantoin, cefuroxime, ampicillin or nystatin (Sigma) were used as a reference antibacterial or antifungal compounds, respectively.

Subsequently, the MIC (Minimal Inhibitory Concentration) of the compounds was examined by the microdilution broth method, using their two-fold dilutions in Mueller-

Hinton broth or (for bacteria) and RPMI 1640 broth with MOPS (for fungi) prepared in 96-well polystyrene plates. Final concentrations of the compounds ranged from 1000 to 0.488 μg/ml. Microbial suspensions were prepared in 0.85% NaCl with an optical density of 0.5 McFarland standard. Next each bacterial or fungal suspension was added per each well containing broth and various concentrations of the examined compounds. After incubation, the MIC was assessed spectrophotometric as the lowest concentration of the samples showing complete bacterial or fungal growth inhibition. Appropriate DMSO, growth and sterile controls were carried out. The medium with no tested substances was used as control.

The MBC (Minimal Bactericidal Concentration) or MFC (Minimal Fungicidal Concentration) are defined as the lowest concentration of the compounds that is required to kill a particular bacterial or fungal species. MBC/MFC was determined by removing the culture using for MIC determinations from each well and spotting onto appropriate agar medium. The plates were incubated under appropriate conditions for bacteria and fungi. The lowest compounds concentrations with no visible growth observed was assessed as a bactericidal/fungicidal concentration. All the experiments were repeated three times and representative data are presented [6,7].

In this study, no bioactivity was defined as a MIC > 1000 μg/mL, mild bioactivity as a MIC in the range 501 – 1000 μg/mL, moderate bioactivity with MIC from 126 to 500 μg/mL, good bioactivity as a MIC in the range 26 – 125 μg/mL, strong bioactivity with MIC between 10 and 25 μg/mL and very strong bioactivity as a MIC < 10 μg/mL [7]. The MBC/MIC or MFC/MIC ratios were calculated in order to determine bactericidal/fungicidal (MBC/MIC ≤ 4, MFC/MIC ≤ 4) or bacteriostatic/fungistatic (MBC/MIC > 4, MFC/MIC > 4) effect of the tested compounds [7].

References:

1. Popiołek, Ł.; Patrejko, P.; Gawrońska-Grzywacz, M.; Biernasiuk, A.; Berecka-Rycerz, A.; Natorska-Chomicka, D.; Piątkowska-Chemiel, I.; Gumieniczek, A.; Dudka, J.; Wujec, M. Synthesis and *in vitro* bioactivity study of new hydrazide-hydrazones of 5-bromo-2-iodobenzoic acid. *Biomed. Pharmacother.* **2020**, 130, 110526, doi: 10.1016/j.biopha.2020.110526
2. Popiołek, Ł.; Rysz, B.; Biernasiuk, A.; Wujec, M. Synthesis of promising antimicrobial agents: hydrazide-hydrazones of 5-nitrofuran-2-carboxylic acid. *Chem. Biol. Drug. Des.* **2020**, 95, 260–269, doi: 10.1111/cbdd.13639
3. Popiołek, Ł.; Tuszyńska, K.; Biernasiuk A. Searching for novel antimicrobial agents among hydrazide-hydrazones of 4-iodosalicylic acid. *Biomed. Pharmacother.* **2022**, 153, 113302, doi: 10.1016/j.biopha.2022.113302
4. European Committee for Antimicrobial Susceptibility Testing (EUCAST) (2003). Determination of minimum inhibitory concentrations (MICs) of antibacterial agents by broth dilution. EUCAST discussion document E. Dis 5.1. *Clin. Microbiol. Infect.* **2003**, *9*, 1-7.
5. Clinical and Laboratory Standards Institute. Reference method for broth dilution antifungal susceptibility testing of yeasts. M27-S4. Clinical and Laboratory Standards Institute, Wayne, PA, USA, **2012**.
6. Wiegand, I.; Hilpert, K.; Hancock, R.E.W. Agar and broth dilution methods to determine the minimal inhibitory concentration (MIC) of antimicrobial substances. *Nat. Protoc.* **2008**, *3(2)*, 163–175.
7. O'Donnell, F.; Smyth T.J.; Ramachandran, V.N.; Smyth, W.F. A study of the antimicrobial activity of selected synthetic and naturally occurring quinolines. *Int. J. Antimicrob. Agents.* **2010**, *35*, 30–38.

Examples of ^{1}H NMR and ^{13}C NMR spectra of synthesized hydrazide-hydrazones of 2,4-dihydroxybenzoic acid

FIGURES

Figure S1. ^{1}H NMR spectrum of compound **1**
Figure S2. ^{13}C NMR spectrum of compound **1**
Figure S3. ^{1}H NMR spectrum of compound **4**
Figure S4. ^{13}C NMR spectrum of compound **4**
Figure S5. ^{1}H NMR spectrum of compound **6**
Figure S6. ^{13}C NMR spectrum of compound **6**
Figure S7. ^{1}H NMR spectrum of compound **8**
Figure S8. ^{13}C NMR spectrum of compound **8**
Figure S9. ^{1}H NMR spectrum of compound **10**
Figure S10. ^{13}C NMR spectrum of compound **10**

International Journal of *Molecular Sciences*

MDPI

Compound **1**: 2,4-dihydroxy-*N*-[(2-methylphenyl)methylidene]benzohydrazide

^{1}H NMR (300 MHz, DMSO-*d*6): 2.45 (s, 3H, CH_3), 6.32-6.40 (m, 2H, ArH), 7.24-7.32 (m, 3H, ArH), 7.80-7.85 (m, 2H, ArH), 8.72 (s, 1H, =CH), 10.24 (s, 1H, OH), 11.69 (s, 1H, NH), 12.43 (s, 1H, OH).

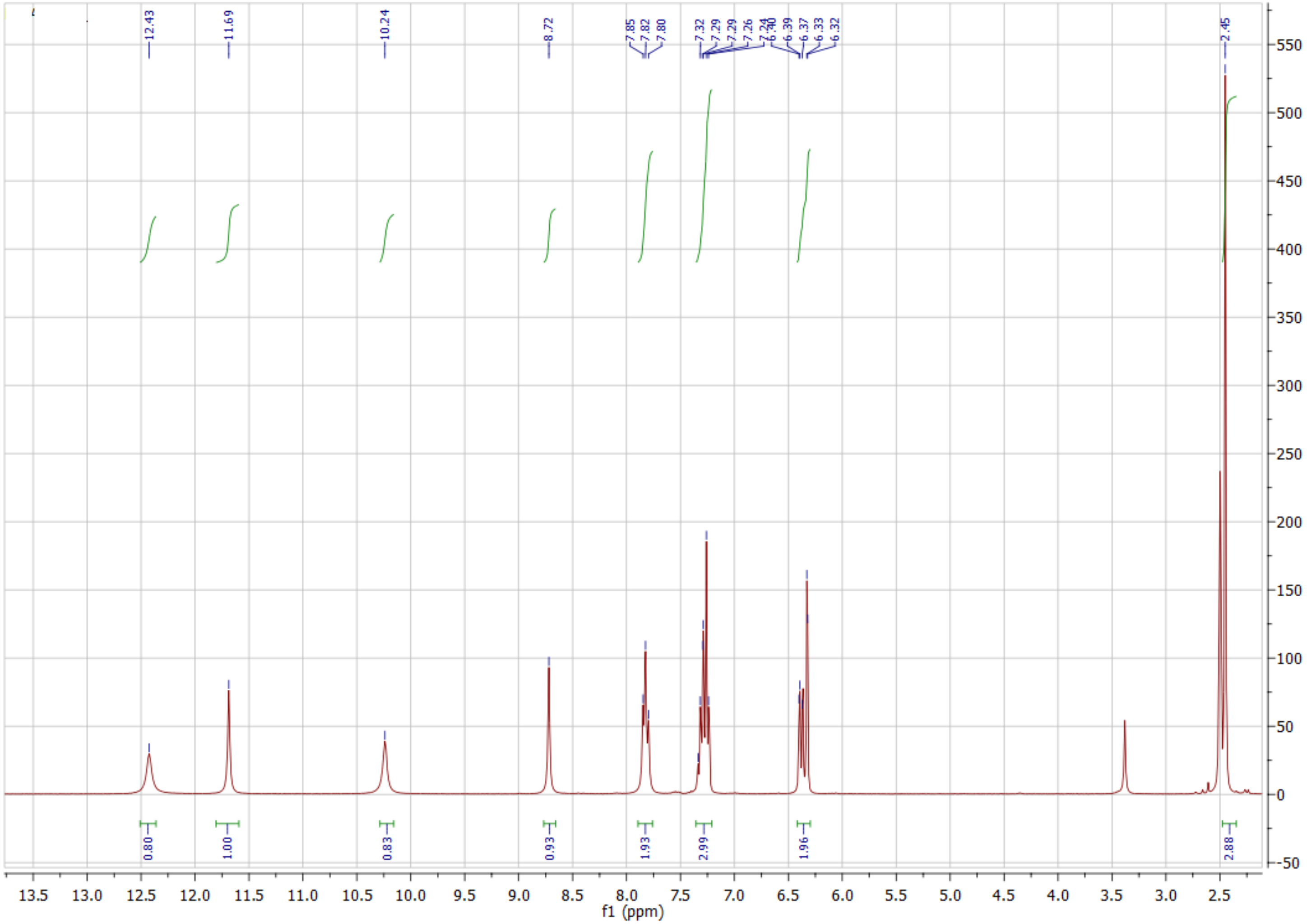

Figure S1. ^{1}H NMR spectrum of compound **1**

Compound **1**: 2,4-dihydroxy-*N*-[(2-methylphenyl)methylidene]benzohydrazide

^{13}C NMR (75 MHz, DMSO-d_6): 19.54 (CH_3), 103.36, 106.49, 107.85, 126.41, 126.65, 129.93, 130.29, 131.35, 132.66, 137.44 (10C_{ar}), 147.15 (=CH), 163.01, 163.22 (2C_{ar}), 166.05 (C=O).

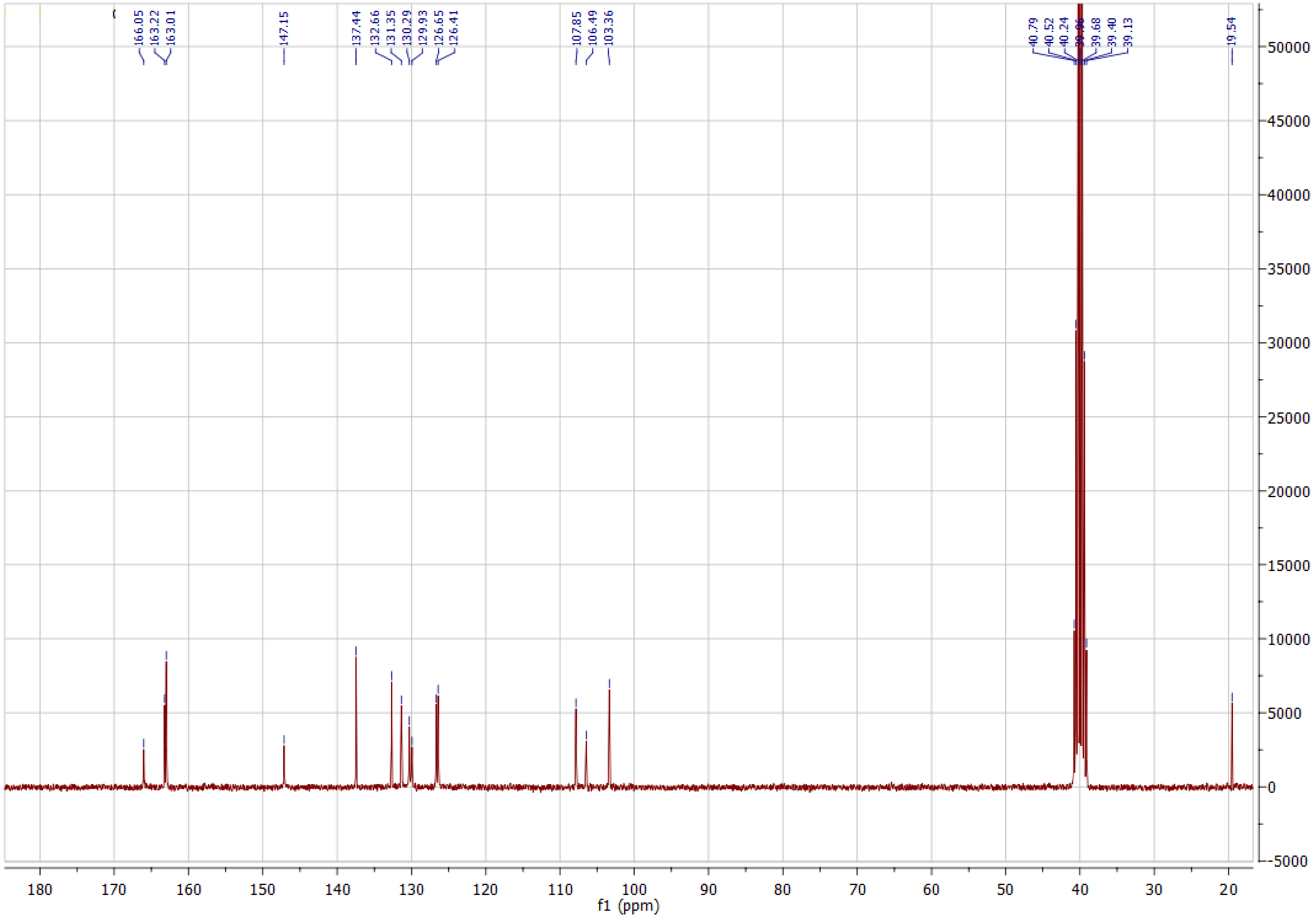

Figure S2. ^{13}C NMR spectrum of compound **1**

Compound **4**: 2,4-dihydroxy-*N*-[(4-methoxyphenyl)methylidene]benzohydrazide

^{1}H NMR (300 MHz, DMSO-d_6): 3.80 (s, 3H, OCH_3), 6.30-6.38 (m, 2H, ArH), 7.01-7.03 (d, 2H, ArH, *J* = 6 Hz), 7.66-7.69 (d, 2H, ArH, *J* = 9 Hz), 7.78-7.81 (d, 1H, ArH, *J* = 9 Hz), 8.37 (s, 1H, =CH), 10.21 (s, 1H, OH), 11.58 (s, 1H, NH), 12.43 (s, 1H, OH).

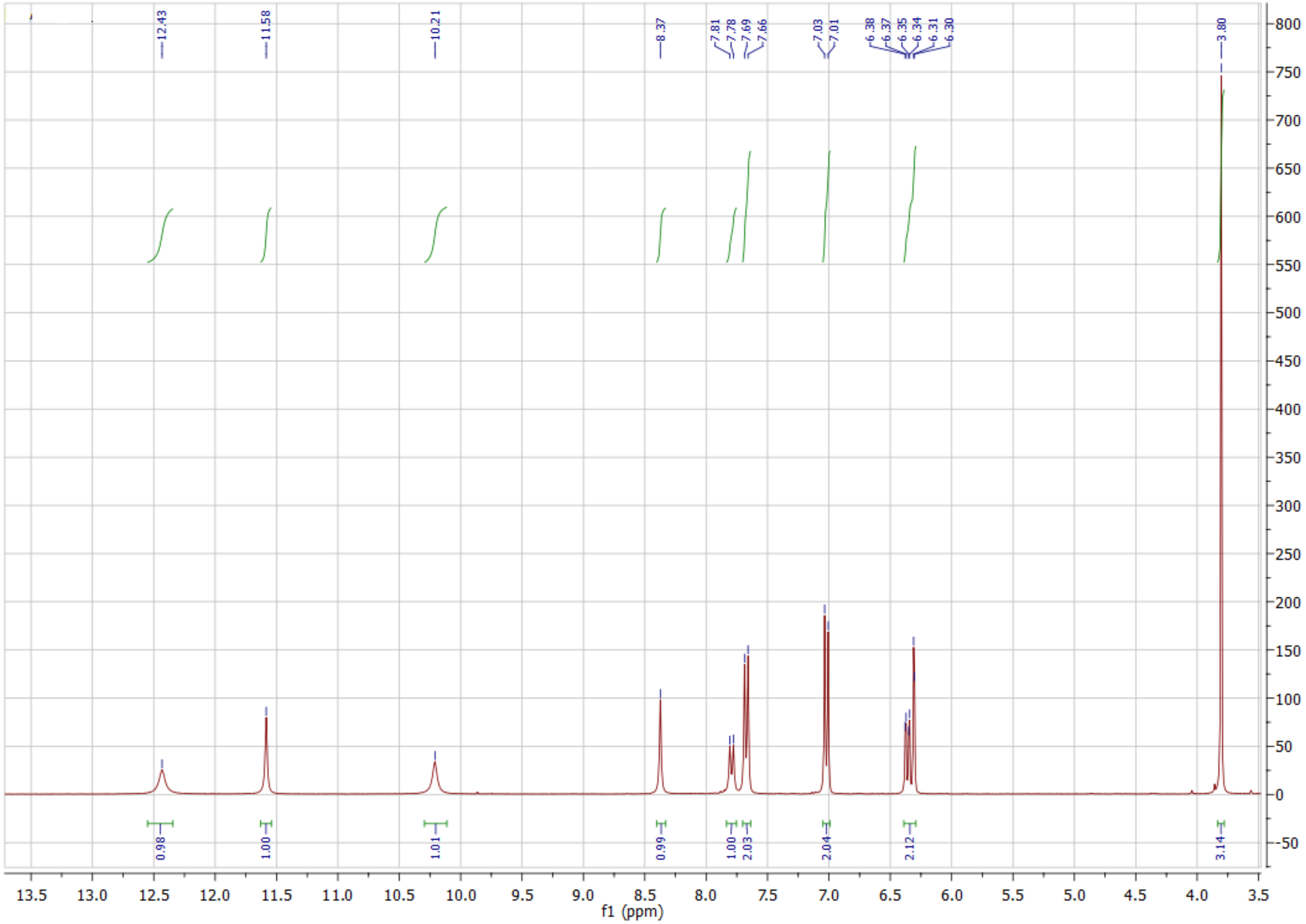

Figure S3. ^{1}H NMR spectrum of compound **4**

Compound **4**: 2,4-dihydroxy-*N*-[(4-methoxyphenyl)methylidene]benzohydrazide

^{13}C NMR (75 MHz, DMSO-d_6): 55.75 (OCH_3), 103.31, 106.65, 107.81, 114.82, 127.21, 129.23, 130.00 (9C_{ar}), 148.39 (=CH), 161.36, 162.78, 163.07 (3C_{ar}), 165.80 (C=O).

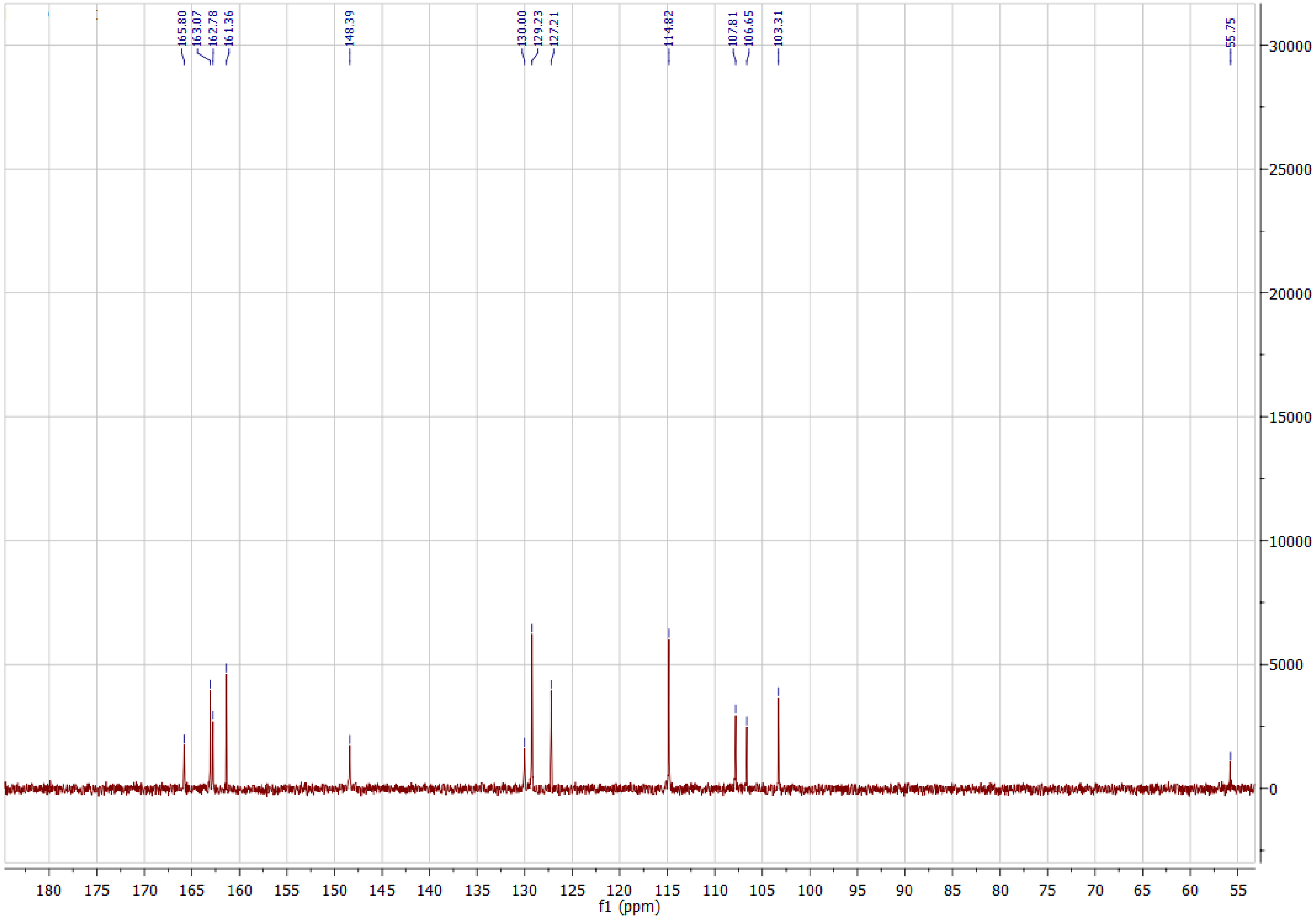

Figure S4. ^{13}C NMR spectrum of compound **4**

Compound **6**: 2,4-dihydroxy-*N*-[(4-propoxyphenyl)methylidene]benzohydrazide

^{1}H NMR (300 MHz, DMSO-d_6): 0.95-1.00 (t, 3H, CH_3, *J* = 9 Hz, *J* = 6 Hz), 1.68-1.80 (m, 2H, CH_2), 3.95-3.99 (t, 2H, CH_2, *J* = 6 Hz), 6.30-6.38 (m, 2H, ArH), 6.99-7.02 (d, 2H, ArH, *J* = 9 Hz), 7.64-7.67 (d, 2H, ArH, *J* = 9 Hz), 7.78-7.81 (d, 1H, ArH, *J* = 9 Hz), 8.37 (s, 1H, =CH), 10.20 (s, 1H, OH), 11.58 (s, 1H, NH), 12.44 (s, 1H, OH).

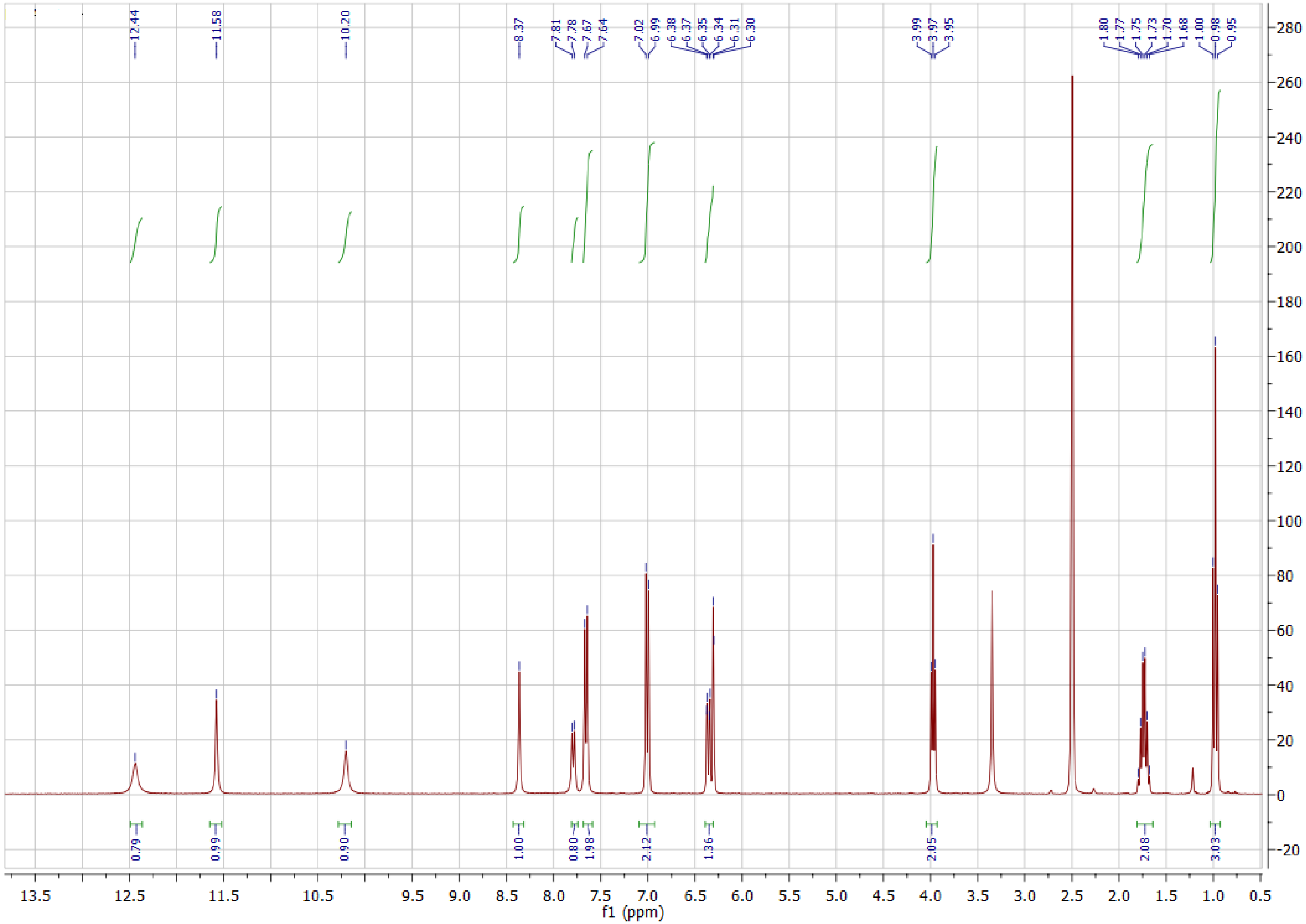

Figure S5. ^{1}H NMR spectrum of compound **6**

Compound **6**: 2,4-dihydroxy-*N*-[(4-propoxyphenyl)methylidene]benzohydrazide

^{13}C NMR (75 MHz, DMSO-*d*$_6$): 10.82 (CH_3), 22.44 (CH_2), 69.57 (CH_2), 103.31, 106.63, 107.80, 115.25, 127.06, 129.24, 129.98 (9C$_{ar}$), 148.40 (=CH), 160.82, 162.80, 163.07 (3C$_{ar}$), 165.81 (C=O).

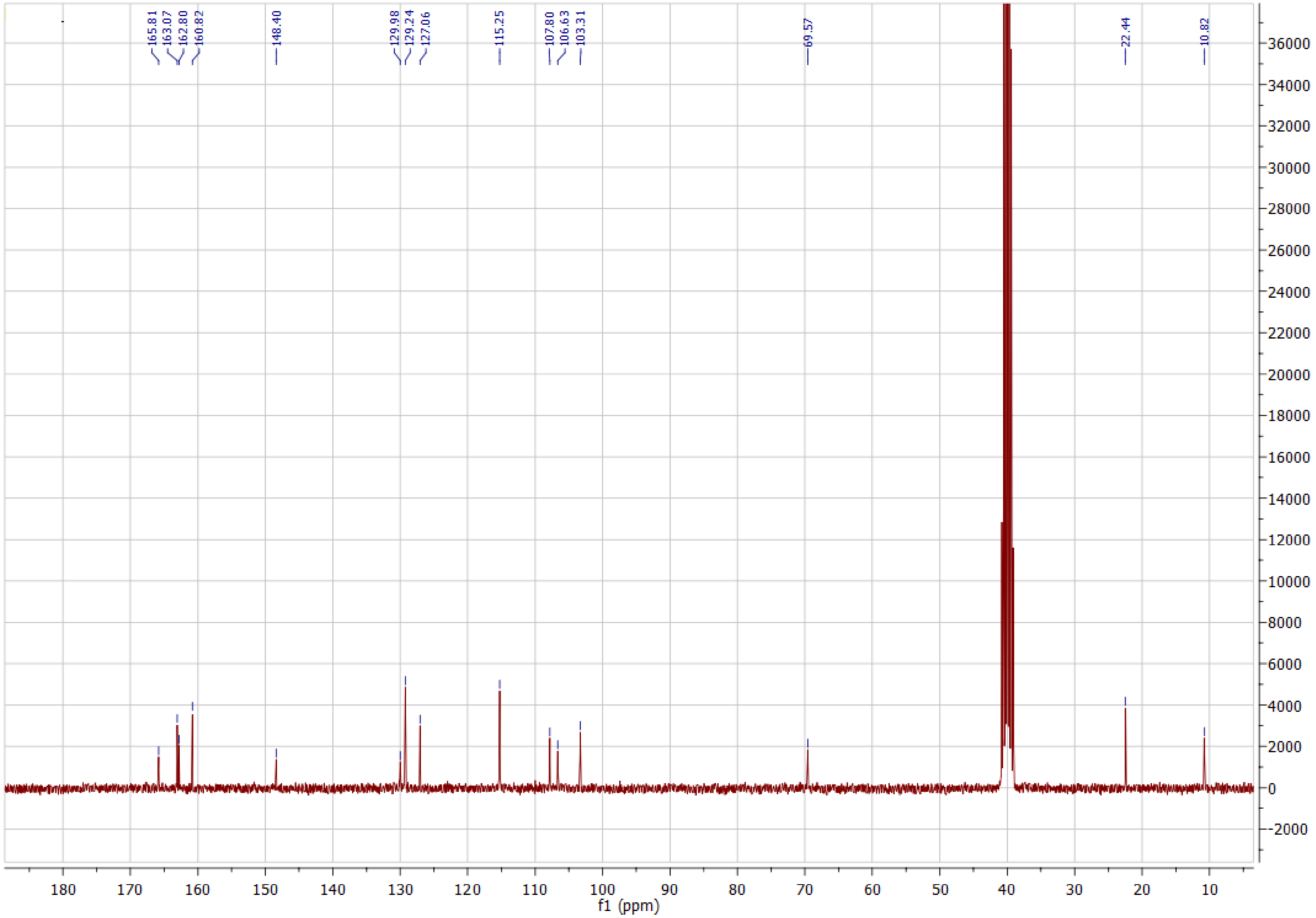

Figure S6. ^{13}C NMR spectrum of compound **6**

Compound **8**: *N*-[(2-chloro-3-methoxyphenyl)methylidene]-2,4-dihydroxybenzohydrazide

^{1}H NMR (300 MHz, DMSO-d_6): 3.88 (s, 3H, OCH_3), 6.31-6.40 (m, 2H, ArH), 7.19-7.21 (m, 1H, ArH), 7.35-7.40 (t, 1H, ArH, *J* = 6 Hz, *J* = 9 Hz), 7.58-7.61 (d, 1H, ArH, *J* = 9 Hz), 7.80-7.83 (d, 1H, ArH, *J* = 9 Hz), 8.86 (s, 1H, =CH), 10.27 (s, 1H, OH), 11.92 (s, 1H, NH), 12.34 (s, 1H, OH).

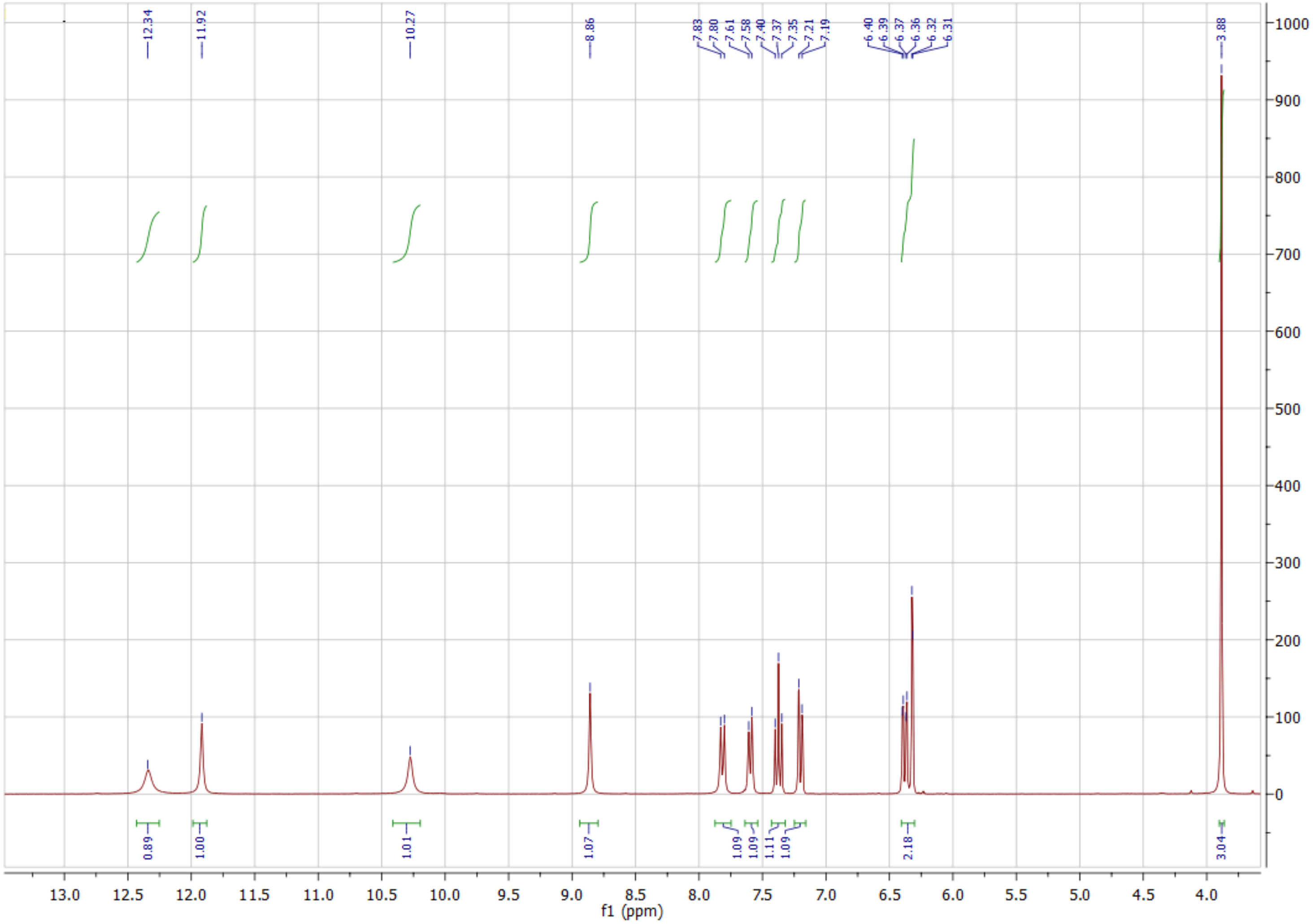

Figure S7. ^{1}H NMR spectrum of compound **8**

Compound **8**: *N*-[(2-chloro-3-methoxyphenyl)methylidene]-2,4-dihydroxybenzohydrazide

^{13}C NMR (75 MHz, DMSO-d_6): 56.77 (OCH_3), 103.34, 106.36, 107.92, 114.11, 118.77, 122.17, 128.31, 130.05, 133.13 (9C_{ar}), 144.61 (=CH), 155.41, 163.13, 163.39 (3C_{ar}), 166.32 (C=O).

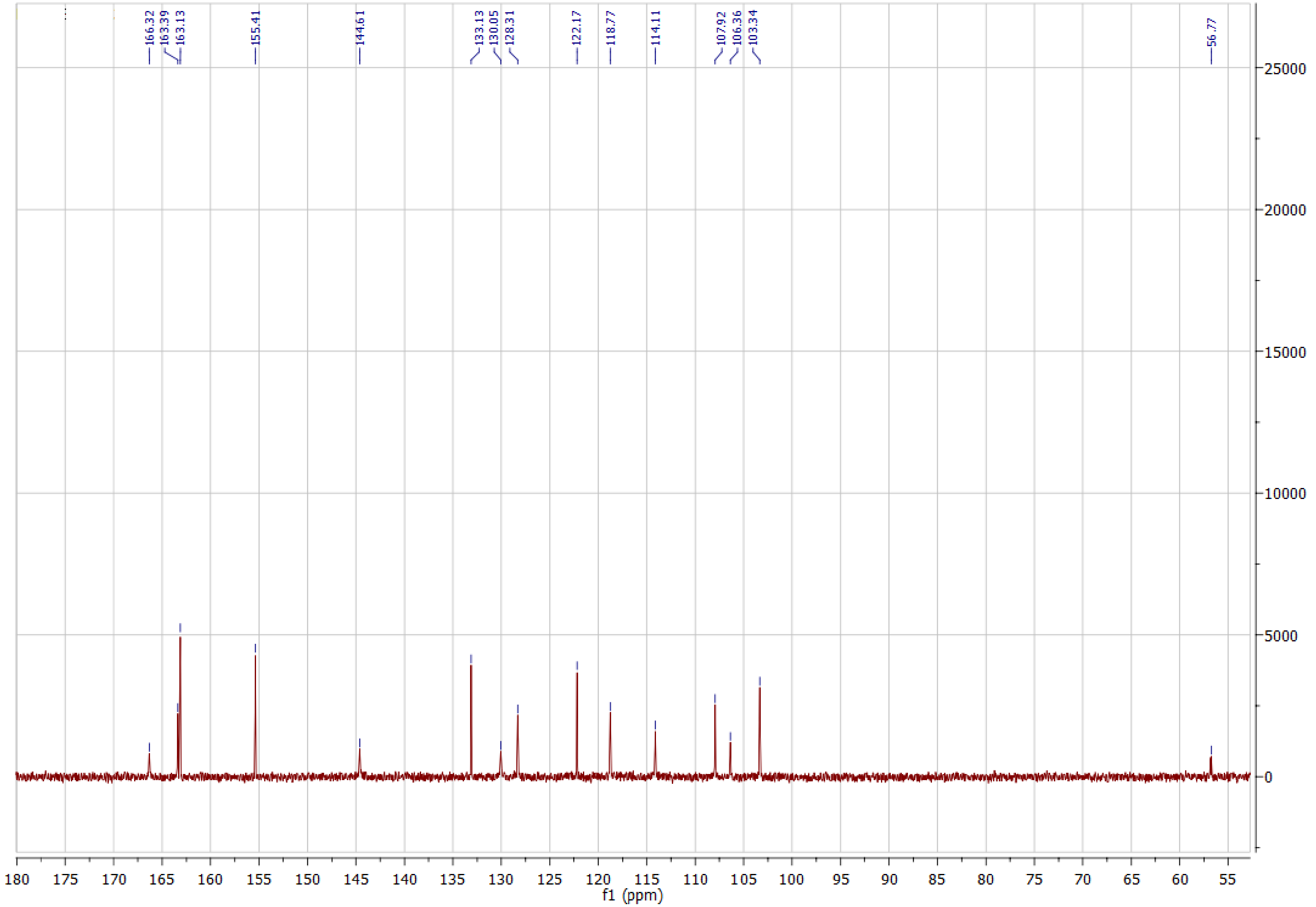

Figure S8. ^{13}C NMR spectrum of compound **8**

Compound **10**: *N*-[(3-bromo-4-methoxyphenyl)methylidene]-2,4-dihydroxybenzohydrazide

^{1}H NMR (300 MHz, DMSO-d_6): 3.90 (s, 3H, OCH_3), 6.32-6.39 (m, 2H, ArH), 7.20-7.23 (d, 1H, ArH, *J* = 9 Hz), 7.67-7.68 (m, 1H, ArH), 7.79-7.82 (m, 2H, ArH), 8.35 (s, 1H, =CH), 10.22 (s, 1H, OH), 11.68 (s, 1H, NH), 12.34 (s, 1H, OH).

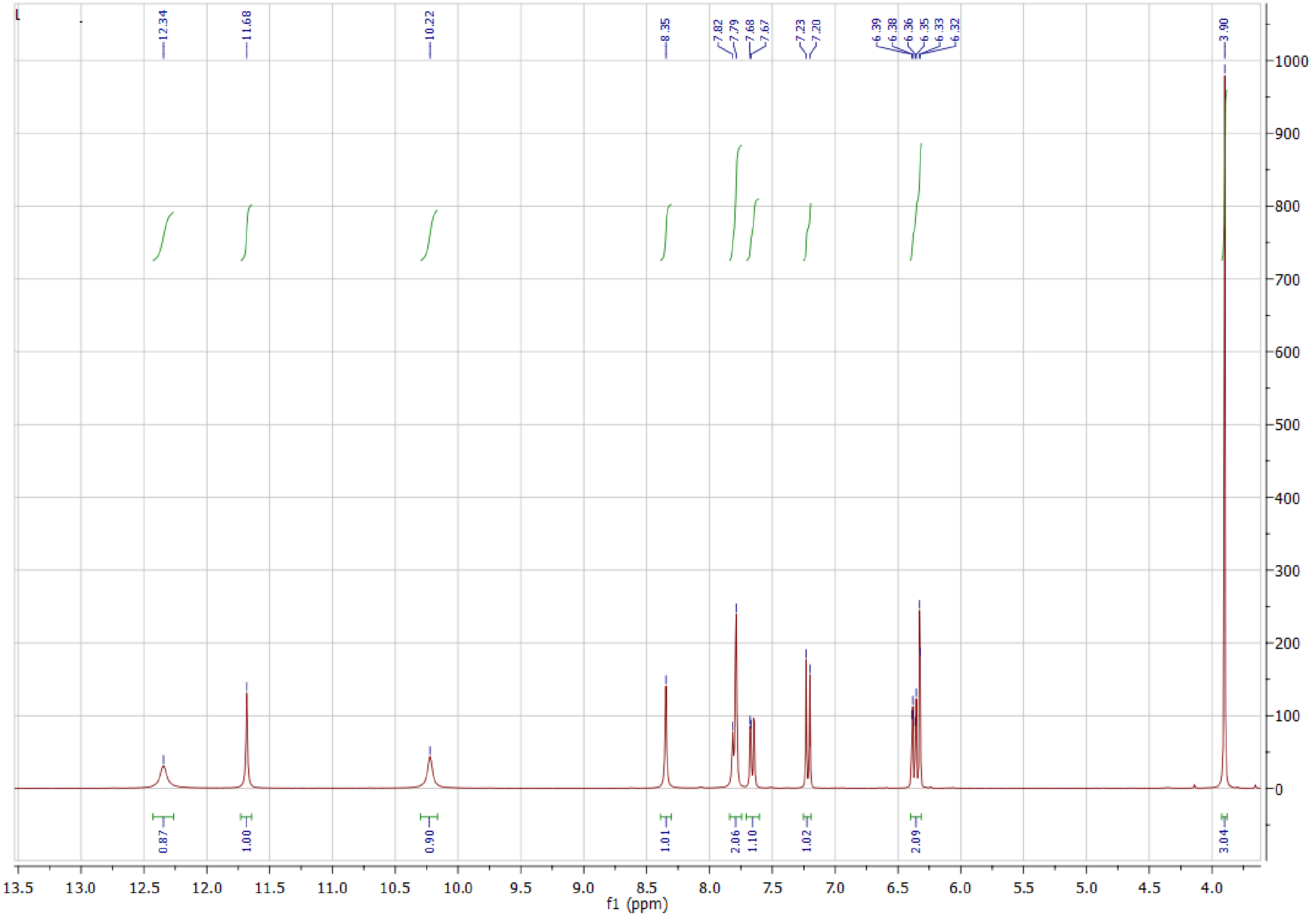

Figure S9. ^{1}H NMR spectrum of compound **10**

Compound **10**: *N*-[(3-bromo-4-methoxyphenyl)methylidene]-2,4-dihydroxybenzohydrazide

^{13}C NMR (75 MHz, DMSO-*d*$_6$): 56.75 (OCH_3), 103.32, 106.69, 107.90, 113.40, 122.14, 128.10, 128.26, 128.36, 133.19 (9C$_{ar}$), 146.83 (=CH), 156.31, 162.66, 163.16 (3C$_{ar}$), 165.84 (C=O).

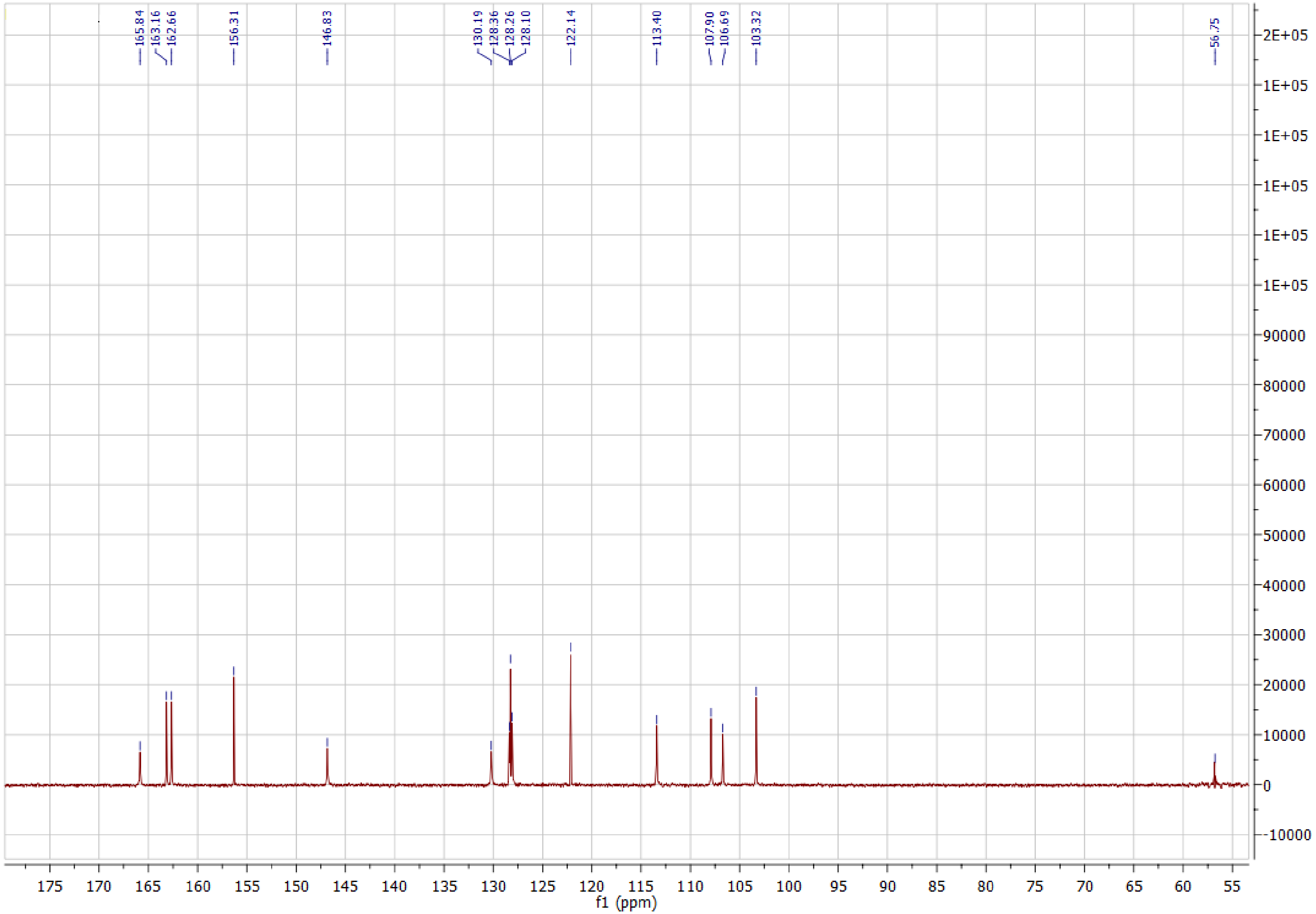

Figure S10. ^{13}C NMR spectrum of compound **10**
